# Supplementary material for: Design of COVID-19 staged alert systems to ensure healthcare capacity with minimal closures
Source: Nat Commun. 2021 Jun 18;12:3767. doi: 10.1038/s41467-021-23989-x (PMC8213780; doi:10.1038/s41467-021-23989-x)
Supplement: Supplementary file 1 — Supplementary Information [file 41467_2021_23989_MOESM1_ESM.pdf]

Supplementary Information for Design of COVID-19 Staged Alert Systems to Ensure Healthcare Capacity with Minimal Closures

Haoxiang Yang, Özge Sürer, Daniel Duque, David P. Morton, Bismark Singh,
Spencer J. Fox, Remy Pasco, Kelly Pierce, Paul Rathouz, Victoria Valencia, Zhanwei Du,
Michael Pignone, Mark E. Escott, Stephen I. Adler, S. Claiborne Johnston, Lauren Ancel Meyers

Supplementary Note 1. Overview

Cities around the world including Austin [1] and Houston [2] in the state of Texas, have staged systems to alert citizens of COVID-19 risks and inform mitigating behavior. Each stage has guidelines for the public, such as opening only essential businesses, avoiding gatherings of specified size, wearing face coverings, and avoiding dining and shopping. Stages with stricter distancing policies reduce disease transmission, but come with a higher socioeconomic cost. We optimize the timing of toggling between stages via triggers that monitor daily new hospital admissions while accounting for total hospitalizations and ICU hospitalizations. Supplementary Method 1 of this supplement gives a detailed description of the stochastic optimization model that we use to derive hospital admissions thresholds for triggering changes between alert stages, including the underlying model of SARS-CoV-2 epidemiological dynamics.

The models we formulate in Supplementary Method 1 include several parameters, which are estimated in four ways: (1) Some parameters are taken from the literature, public sources, or from city officials in Austin and Houston as detailed in Supplementary Table 10. (2) Other key parameters governing COVID-19 patient hospital and ICU durations are directly estimated from data provided by a multi-facility hospital system serving the central Texas region, including Austin, Texas ("Hospital system data"). These parameters are summarized in Supplementary Method 2 and Supplementary Table 11, and described in detail in ref. [3]. (3) We estimate the transmission-reduction caused by various control measures by least-squares fitting of the model to COVID-19 hospitalization time-series data from Austin and Houston during different pandemic periods, as described in Supplementary Method 2. (4) There are four macro-stochastic parameters that govern the sojourn time in the exposed compartment of our SEIR-style model, the recovery times from the asymptomatic and symptomatic compartments, and the infectiousness of an asymptomatic individual relative to an infectious individual. We postulate triangular distributions for each of these four parameters (Supplementary Table 11), and use a Monte Carlo procedure to effectively derive a posterior joint distribution for these four parameters that is consistent with observed hospitalizations. This procedure and the resulting distributions are described in Supplementary Method 3.

Supplementary Discussion 1 provides four analyses: (1) a derivation of a staged alert system for Houston, Texas following the method presented in the main manuscript; (2) a derivation of optimal triggers for launching an alternate care site (i.e., field hospital), should existing hospital capacity prove inadequate; (3) a sensitivity analysis regarding public compliance with recommended policies; (4) a retrospective assessment of the staged alert system guiding COVID-19 responses in Austin, Texas from May 2020 through April 2021. Supplementary Discussion 2 reviews our experience with Austin's staged alert system after September 2020.

Supplementary Method 1. Epidemiological and Optimization Model

The mathematical optimization model that we propose recommends a stricter stage of lock-down when the seven-day moving average of daily hospital admissions grows to exceed the stage's optimized *threshold*. A more relaxed stage is recommended when the same moving average drops below the stage's threshold. The sojourn time in each stage must be at least two weeks. We call this the "trigger policy."

To find the thresholds for each stage, we form a stochastic optimization model, in which disease dynamics are characterized by an enhanced SEIR-style model of disease transmission [4]. This epidemiological model comprises compartments for susceptible, exposed, pre-asymptomatic, pre-symptomatic, infectious-asymptomatic, infectious-symptomatic, infected and hospitalized in the general ward, infected and hospitalized in the intensive care unit (ICU), recovered, and deceased, which we denote by S, E, PA, PY, IA, IY, IH, ICU, R, and D, respectively. Supplementary Figure 1 diagrams the model's compartments and transitions for a metropolitan area. The population is partitioned into ten groups consisting of five age groups of low-risk and another five of high-risk individuals. Each group is represented with its own set of ten compartments (susceptible, exposed, etc.) so that in total the epidemiological model has 100 compartments.

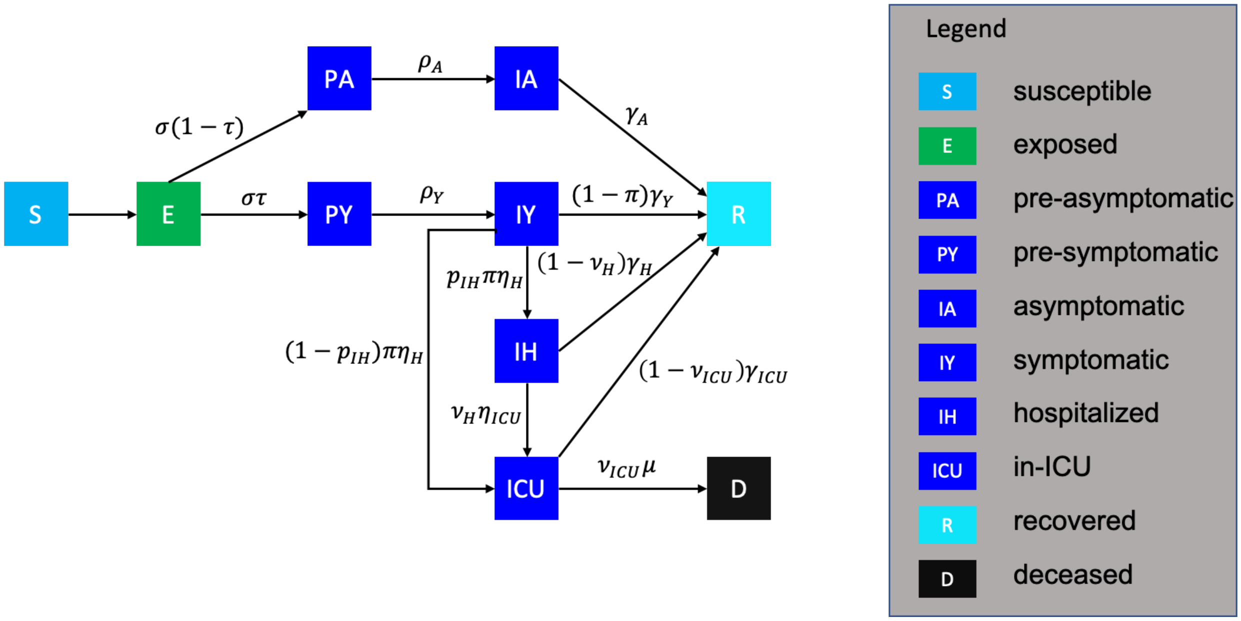

Supplementary Figure 1: Diagram of the epidemiological model with compartments, transitions, and rates, with definitions provided in the enumerated notation that follows. The model has ten copies of such diagrams for each age-risk group pair, which interact to determine the (unmarked) rate of transition from compartment S to E. Such transitions are governed by the transmission rate β but also contact matrices and the current risk stage.

We assume that the threshold for each stage is constant over time and is denoted by ℓ_i for stage i. The goal is to determine level ℓ_i so that the total expected cost of our trigger policy is minimized, while respecting epidemiological dynamics and hospital capacity using a probabilistic constraint. We approximate the expectation and probabilistic constraint by using Monte Carlo simulation to form a set of scenarios, indexed by ω, and by using binary variables to count the number of paths that violate capacity and to determine the stage for each sample path at each point in time.

<u>Notation:</u>

Indices and Sets

$t \in \mathcal{T}$ set of time periods $\{1, 2, \ldots, |\mathcal{T}|\}$ [day]

$t \in \mathcal{T}_0$ $\mathcal{T} \cup \{0\}$

$a \in \mathcal{A}$ set of age groups {0-4y, 5-17y, 18-49y, 50-64y, 65y+}
$r \in \mathcal{R}$ risk groups $\{low, high\}$
$i \in \mathcal{I}$ predefined stages $\{1\,\text{(red)}, 2\,\text{(orange)}, 3\,\text{(yellow)}, 4\,\text{(blue)}\}$
$\omega \in \Omega$ set of scenarios

Parameters
Epidemiological parameters:

β unmitigated transmission rate
σ rate at which exposed individuals become infectious
τ proportion of exposed individuals who become symptomatic
ρ_A rate at which pre-asymptomatic individuals become asymptomatic
ρ_Y rate at which pre-symptomatic individuals become symptomatic
γ_A recovery rate from asymptomatic compartment
γ_Y recovery rate from symptomatic compartment
γ_H^a recovery rate from hospitalized compartment for age group a
γ_{ICU}^a recovery rate from ICU compartment for age group a
P proportion of pre-symptomatic transmission
$YHR^{a,r}$ percent of symptomatic infectious that go to the hospital for age-risk group a, r
η_H hospitalization rate after symptom onset
ω_A infectiousness of individuals in IA relative to IY
$\omega_P^{a,r}$ $\frac{P}{1-P}\frac{\tau(YHR^{a,r}/\eta_H+(1-YHR^{a,r})/\gamma_Y)+(1-\tau)\omega_A/\gamma_A}{\tau/\rho_Y+(1-\tau)\omega_A/\rho_A}$: infectiousness of pre-symptomatic individuals relative to IY for age-risk group a, r
$\pi^{a,r}$ $\frac{\gamma_Y \cdot YHR^{a,r}}{[\eta_H-(\eta_H-\gamma_Y)YHR^{a,r}]}$: rate-adjusted proportion of symptomatic individuals who go to the hospital for age-risk group a, r
p_{IH} percent of patients directly going to the general ward of the hospital
$HICUR$ percent of general ward patients who get transferred to ICU
η_{ICU}^a ICU admission rate after admission to the general ward for age group a
ν_H^a $\frac{\gamma_H^a \cdot HICUR}{[\eta_{ICU}^a-(\eta_{ICU}^a-\gamma_H^a)HICUR]}$: rate-adjusted proportion of general ward patients transferred to ICU for age group a
μ^a rate from ICU to death for age group a
$ICUFR^a$ percent of hospitalized that die for age group a
ν_{ICU}^a $\frac{\gamma_{ICU}^a \cdot ICUFR^a}{[\mu^a-(\mu^a-\gamma_{ICU}^a)ICUFR^a]}$: ICU fatality rate-adjusted proportion for age group a
$\phi_{i,t}^{a',r',a,r}$ expected number of daily contacts from (a', r') to (a, r) at time t under stage i
$N^{a,r}$ population of age-risk group a, r

Additional parameters:

B number of hospital beds, including general ward and ICU, for COVID-19 patients
B_{ICU} number of ICU beds for COVID-19 patients
ε violation probability for probabilistic constraint
C_i daily cost of being in stage i

Variables
Epidemiological variables (for scenario $\omega \in \Omega$):

$S_{t,\omega}^{a,r}$ number of susceptible people of age group a, risk group r at time t [persons]
$dS_{t,\omega}^{a,r}$ $S_{t,\omega}^{a,r} - S_{t+1,\omega}^{a,r}$ [persons]
$E_{t,\omega}^{a,r}$ number of exposed people of age group a, risk group r at time t [persons]
$PA_{t,\omega}^{a,r}$ number of pre-asymptomatic people for a, r, t [persons]
$PY_{t,\omega}^{a,r}$ number of pre-symptomatic people for a, r, t [persons]
$IA_{t,\omega}^{a,r}$ number of infectious-asymptomatic people for a, r, t [persons]
$IY_{t,\omega}^{a,r}$ number of infectious-symptomatic people for a, r, t [persons]
$IH_{t,\omega}^{a,r}$ number of infected-hospitalized people in the general ward for a, r, t [persons]

$ICU^{a,r}_{t,\omega}$ number of infected-hospitalized people in the ICU for a, r, t [persons]
$R^{a,r}_{t,\omega}$ number of recovered people for a, r, t [persons]
$D^{a,r}_{t,\omega}$ number of deceased people for a, r, t [persons]
$H_{t,\omega}$ daily hospital admissions, from infectious-symptomatic to the general ward and ICU, at time t [persons/day]
$\overline{H}_{t,\omega}$ seven-day moving average of $H_{t,\omega}$ [persons/day]
$U_{t,\omega}$ daily ICU admissions (from infectious-symptomatic and the general ward) at time t [persons/day]

Intervention variables:

ℓ_i the stage-specific threshold (level) for the daily hospitalization rate
$X_{i,t,\omega}$ 1 if the system is in stage i at time t for scenario ω; 0 otherwise
$Y_{i,t,\omega}$ 1 if the trigger statistic lies in range of stage i at time t for scenario ω; 0 otherwise
$V_{i,t,\omega}$ 1 if $Y_{i,t,\omega} = 1$ prior to spending 14 days in the previous stage for scenario ω; 0 otherwise
Z_ω 1 if healthcare capacity is exceeded in scenario ω; 0 otherwise

We refer to Supplementary Table 10 for further details on model parameters. We first define the epidemiological transition dynamics in the following equations for all $\omega \in \Omega$. These dynamics largely follow the formulation used in [5] with the addition of three compartments to improve model fidelity and to distinguish beds in the ICU and general ward. The initial conditions specify a single infectious individual in the 18-49 age group with low risk. The age-risk groups are initialized with the rest of the population in their respective susceptible compartments. Eqs. [1a]-[1m] below then provide a sample path, indexed by ω, for the progression of the disease in the community. For the moment, the indicator variables $X_{i,t,\omega} \in \{0, 1\}$ are taken as input, and select the current stage and, in turn, the expected number of daily contacts via $\phi^{a',r',a,r}_{i,t}$. The contact matrices are indexed by t because they capture whether school is currently open and if so, the school calendar; they further capture weekdays versus weekends and the level of cocooning, which can vary with time; and they capture contacts at school, home, work, and another catch-all category. We assume that sufficient precautions are taken in hospitals so that hospitalized cases do *not* contribute to infecting others via Eq. [1m]. The most significant updates of the model from that in [5] are in additional compartments. We use constructs similar to He et al. [6] for a pre-symptomatic period to more accurately model the profile of infectiousness of individuals by including pre-symptom onset transmission. We also model the ICU compartment explicitly for two reasons. First, patients in the ICU have different durations in the hospital than those in the general ward, and second it allows us to account for ICU capacity as a resource. We let p_{IH} denote the probability a hospitalized patient is admitted to a general ward bed and the remaining fraction go directly to the ICU. As Supplementary Figure 1 and Eq. [1h] indicate, it is possible to transfer general ward patients to the ICU later if needed. All deaths are recorded from the ICU.

For simplicity, we write the finite-difference Eqs. [1] in a deterministic form. They become stochastic, and require indexing by ω, because binomial random variables replace terms like $\sigma E^{a,r}_{t,\omega}$; here the binomial random variable has parameter $n = E^{a,r}_{t,\omega}$ and σ serves as the "success" probability. This construct is pervasive throughout right-hand side terms in Eqs. [1]. In addition to these "micro" stochastics there are "macro" stochastics because we model σ, ω_A, γ_A, and γ_Y as random variables that are subject to a Monte Carlo draw at time 0 of the simulation. We discuss this further

in Supplementary Method 3.

$$
\begin{aligned}
S^{a,r}_{t+1,\omega} - S^{a,r}_{t,\omega} =& - dS^{a,r}_{t,\omega} && \forall t \in \mathcal{T}_0, a \in \mathcal{A}, r \in \mathcal{R} && \text{[1a]}\\
E^{a,r}_{t+1,\omega} - E^{a,r}_{t,\omega} =& dS^{a,r}_{t,\omega} - \sigma E^{a,r}_{t,\omega} && \forall t \in \mathcal{T}_0, a \in \mathcal{A}, r \in \mathcal{R} && \text{[1b]}\\
PA^{a,r}_{t+1,\omega} - PA^{a,r}_{t,\omega} =& \sigma(1-\tau)E^{a,r}_{t,\omega} - \rho_A PA^{a,r}_{t,\omega} && \forall t \in \mathcal{T}_0, a \in \mathcal{A}, r \in \mathcal{R} && \text{[1c]}\\
IA^{a,r}_{t+1,\omega} - IA^{a,r}_{t,\omega} =& \rho_A PA^{a,r}_{t,\omega} - \gamma_A IA^{a,r}_{t,\omega} && \forall t \in \mathcal{T}_0, a \in \mathcal{A}, r \in \mathcal{R} && \text{[1d]}\\
PY^{a,r}_{t+1,\omega} - PY^{a,r}_{t,\omega} =& \sigma\tau E^{a,r}_{t,\omega} - \rho_Y PY^{a,r}_{t,\omega} && \forall t \in \mathcal{T}_0, a \in \mathcal{A}, r \in \mathcal{R} && \text{[1e]}\\
IY^{a,r}_{t+1,\omega} - IY^{a,r}_{t,\omega} =& \rho_Y PY^{a,r}_{t,\omega} - (1-\pi^{a,r})\gamma_Y IY^{a,r}_{t,\omega} - \pi^{a,r}\eta_H IY^{a,r}_{t,\omega} && \forall t \in \mathcal{T}_0, a \in \mathcal{A}, r \in \mathcal{R} && \text{[1f]}\\
IH^{a,r}_{t+1,\omega} - IH^{a,r}_{t,\omega} =& p_{IH}\pi^{a,r}\eta_H IY^{a,r}_{t,\omega} - (1-\nu^a_H)\gamma^a_H IH^{a,r}_{t,\omega} - \nu^a_H \eta^a_{ICU} IH^{a,r}_{t,\omega} && \forall t \in \mathcal{T}_0, a \in \mathcal{A}, r \in \mathcal{R} && \text{[1g]}\\
ICU^{a,r}_{t+1,\omega} - ICU^{a,r}_{t,\omega} =& (1-p_{IH})\pi^{a,r}\eta_H IY^{a,r}_{t,\omega} + \nu^a_H \eta^a_{ICU} IH^{a,r}_{t,\omega} - && && \text{[1h]}\\
& (1-\nu^a_{ICU})\gamma^a_{ICU} ICU^{a,r}_{t,\omega} - \nu^a_{ICU}\mu^a ICU^{a,r}_{t,\omega} && \forall t \in \mathcal{T}_0, a \in \mathcal{A}, r \in \mathcal{R} && \text{[1i]}\\
R^{a,r}_{t+1,\omega} - R^{a,r}_{t,\omega} =& \gamma_A IA^{a,r}_{t,\omega} + (1-\pi^{a,r})\gamma_Y IY^{a,r}_{t,\omega} + (1-\nu^a_H)\gamma^a_H IH^{a,r}_{t,\omega} + && && \text{[1j]}\\
& (1-\nu^a_{ICU})\gamma^a_{ICU} ICU^{a,r}_{t,\omega} && \forall t \in \mathcal{T}_0, a \in \mathcal{A}, r \in \mathcal{R} && \text{[1k]}\\
D^{a,r}_{t+1,\omega} - D^{a,r}_{t,\omega} =& \nu^a_{ICU}\mu^a ICU^{a,r}_{t,\omega} && \forall t \in \mathcal{T}_0, a \in \mathcal{A}, r \in \mathcal{R} && \text{[1l]}\\
dS^{a,r}_{t,\omega} =& S^{a,r}_{t,\omega} \sum_{a' \in \mathcal{A}} \sum_{r' \in \mathcal{R}} \sum_{i \in \mathcal{I}} \frac{\beta \phi^{a',r',a,r}_{i,t} X_{i,t,\omega}}{N^{a',r'}} \Big(IY^{a',r'}_t + \omega_A IA^{a',r'}_t + && && \\
& \omega^{a',r'}_P \omega_A PA^{a',r'}_t + \omega^{a',r'}_P PY^{a',r'}_t \Big) && \forall t \in \mathcal{T}_0, a \in \mathcal{A}, r \in \mathcal{R}. && \text{[1m]}
\end{aligned}
$$

The initial conditions, for analysis in both Austin and Houston, have all variables indexed by $t = 0$ as zero except the following:

$$IY^{18\text{-}49,low}_{0,\omega} = 1, S^{18\text{-}49,low}_{0,\omega} = N^{18\text{-}49,low} - 1, \text{and } S^{a,r}_{0,\omega} = N_{a,r} \forall (a,r) \in \mathcal{A} \times \mathcal{R} \setminus \{(18\text{-}49, low)\}. \qquad [2]$$

The stochastic optimization model is formulated as we detail next. For each sample path, ω, we record the number of daily hospital admissions and its seven-day moving average in constraints [3c] and [3d], aggregated across the general ward and ICU. At time t, the stage is decided using two criteria. We are nominally in stage i if the trigger statistic lies between the threshold of stage i, ℓ_i, and that of stage $i+1$, ℓ_{i+1}. This occurs unless we would spend fewer than 14 days in our current stage, in which case we cannot yet switch stages. This logic is enforced by constraints [3e]-[3j]. Constraint [3e] identifies the would-be stage based on the trigger statistic, which is the seven-day moving average of the hospital admissions. The big-M coefficient in constraint [3e] stands for a sufficiently large number. Constraint [3f] forces variable $V_{i,t,\omega}$ to take value 1 if we switched to stage i in the previous 14 days and value 0 otherwise, and thus it forces the system to stay in the same stage through constraint [3g]. If $V_{i,t,\omega} = 0$ then constraint [3h] means that the stage is decided by the trigger statistic through variable $Y_{i,t,\omega}$. Constraint [3i] states that only one stage can be selected at any time-scenario (t,ω) pair.

The expected cost is calculated in the objective function [3a] by summing the daily socioeconomic costs over the time horizon. Constraint [3k] identifies scenarios for which, at some time point, the number of ICU patients exceeds the ICU capacity, where M is again sufficiently large so the constraint is vacuous if $Z_\omega = 1$. Constraint [3l] ensures the probability such a violation occurs is at most fraction ε of the total scenarios. Here we use the number of ICU patients in the probabilistic constraint, but it can be replaced by the number of total hospitalized patients, respectively replacing $ICU^{a,r}_{t,\omega}$ and B_{ICU} by $ICU^{a,r}_{t,\omega} + IH^{a,r}_{t,\omega}$ and B.

We have formulated the model with daily time periods, which simplifies notation for computing the seven-day moving average of new admissions, the logic behind the rule of spending two weeks in a stage, etc. That said, in implementation we use ten time steps per day, which suffices for the fidelity of the epidemiological dynamics in Eqs. [1].

We generate 300 scenarios for Ω according to the procedure described in Supplementary Method 3. After obtaining a set of optimal triggers, we generate another 300 scenarios, Ω', to assess performance. Those two sets of scenarios are similar to "training" and "testing" data used in statistics and machine learning. As a result, it is possible to obtain

thresholds that meet the probabilistic constraint under Ω, but violate that constraint when tested using Ω', although in our experience such violations are both rare and modest.

Model [3] is a large-scale stochastic mixed-integer nonlinear program. Problems of the scale we consider cannot currently be solved using commercial integer programming software. We approximately solve the model using a grid search procedure. For a fixed set of thresholds, $\ell_i, i \in \mathcal{I}$, which satisfy inequalities [3m], we can run the simulation model for all $\omega \in \Omega$ in parallel, applying transition dynamics [1]-[2] and using the logic of [3c]-[3k] and [3n]-[3o] to compute the binary variables which indicate the stages, $X_{i,t,\omega}$, and the scenarios with capacity violations, Z_ω. For each set of thresholds that we consider in our grid search, we select the configuration that yields the minimum expected cost [3a] while satisfying the probabilistic constraint [3l]. Our grid search considers all configurations of the thresholds $\ell_i, i \in \mathcal{I}$, using increments of 10 that satisfy [3m] after computing an upper bound on ℓ_i necessary to satisfy the probabilistic constraint [3l].

$$
\begin{aligned}
\min \quad & \frac{1}{|\Omega|} \sum_{\omega \in \Omega} \sum_{i \in \mathcal{I}} \sum_{t \in \mathcal{T}} C_i X_{i,t,\omega} & & \text{[3a]} \\
\text{s.t.} \quad & \text{Equations [1] and [2]} & & \text{[3b]} \\
& H_{t,\omega} = \sum_{a \in \mathcal{A}} \sum_{r \in \mathcal{R}} \pi^{a,r} \eta_H IY^{a,r}_{t,\omega} & \forall t \in \mathcal{T}, \omega \in \Omega & \quad \text{[3c]} \\
& \overline{H}_{t,\omega} = \frac{1}{7} \sum_{t'=\max\{t-6,1\}}^{t} H_{t,\omega} & \forall t \in \mathcal{T}, \omega \in \Omega & \quad \text{[3d]} \\
& \ell_i - M(1 - Y_{i,t,\omega}) \leq \overline{H}_{t,\omega} \leq \ell_{i+1} + M(1 - Y_{i,t,\omega}) & \forall i \in \mathcal{I}, t \in \mathcal{T}, \omega \in \Omega & \quad \text{[3e]} \\
& 2V_{i,t,\omega} - 1 \leq X_{i,t-1,\omega} - X_{i,\max\{t-14,0\},\omega} \leq V_{i,t,\omega} & \forall i \in \mathcal{I}, t \in \mathcal{T}, \omega \in \Omega & \quad \text{[3f]} \\
& X_{i,t,\omega} \geq V_{i,t,\omega} & \forall i \in \mathcal{I}, t \in \mathcal{T}, \omega \in \Omega & \quad \text{[3g]} \\
& X_{i,t,\omega} \leq Y_{i,t,\omega} + V_{i,t,\omega} & \forall i \in \mathcal{I}, t \in \mathcal{T}, \omega \in \Omega & \quad \text{[3h]} \\
& \sum_{i \in \mathcal{I}} X_{i,t,\omega} = 1 & \forall t \in \mathcal{T}, \omega \in \Omega & \quad \text{[3i]} \\
& X_{i,0,\omega} = 0 & \forall i \in \mathcal{I}, \omega \in \Omega & \quad \text{[3j]} \\
& \sum_{a \in \mathcal{A}} \sum_{r \in \mathcal{R}} ICU^{a,r}_{t,\omega} \leq B_{ICU} + MZ_\omega & \forall t \in \mathcal{T}, \omega \in \Omega & \quad \text{[3k]} \\
& \sum_{\omega \in \Omega} Z_\omega \leq \lfloor \varepsilon |\Omega| \rfloor & & \quad \text{[3l]} \\
& 0 \leq \ell_i \leq \ell_{i-1} & \forall i \in \mathcal{I} \setminus \{1\} & \quad \text{[3m]} \\
& X_{i,t,\omega}, V_{i,t,\omega}, Y_{i,t,\omega} \in \{0,1\} & \forall i \in \mathcal{I}, t \in \mathcal{T}, \omega \in \Omega & \quad \text{[3n]} \\
& Z_\omega \in \{0,1\} & \forall \omega \in \Omega. & \quad \text{[3o]}
\end{aligned}
$$

Supplementary Method 2. Model Parameters

The cost coefficients C_1, C_2, C_3, and C_4 are important parameters in the optimization model [3]. The foremost driver in the probabilistic constraint requires that we respect ICU capacity. Policies that satisfy that constraint specify a set of allowable thresholds. We choose $C_1 \gg C_2 \gg C_3 \gg C_4$ so that the resulting policy is "hierarchical." Effectively, the model first tries to find policies that minimize expected time in stage 1 and satisfy the probabilistic constraint on ICU capacity. Among those it tries to minimize the expected time spent in stage 2, etc. For our specific problem instances, we first computed results with $C_1 = 10^3$, $C_2 = 10^2$, $C_3 = 10^1$, and $C_4 = 10^0$. Then we solved the model again with $C_1 = 10^6$, $C_2 = 10^4$, $C_3 = 10^2$, and $C_4 = 10^0$. The obtained optimal thresholds and the detailed results are identical in both cases and correspond to what we report in Table 2 in the main text. This suggests that the factor of 10 for C_i/C_{i+1} is sufficiently large to induce the desired hierarchical property. This overall cost structure represents the

preference of avoiding increasingly strident alert levels, and has been proven effective in the City of Austin in practice, as discussed in the main manuscript and as further detailed in Supplementary Discussion 1.

Supplementary Tables 1 and 2 partition the population of the Austin and Houston MSAs based on age groups (0-4 years old, 5-17 years old, 18-49 years old, 50-64 years old, and 65 years and older) and risk groups (low risk and high risk). The high-risk group proportions are estimated based on the population with chronic conditions listed by the CDC 500 cities data [7]. Population data processing is detailed in the appendix of [5] and here we present only the final numbers used for this paper's analysis.

$N^{a,r}$	0-4	5-17	18-49	50-64	65 and older
Low risk	128527	327148	915894	249273	132505
High risk	9350	37451	156209	108196	103763

Supplementary Table 1: Austin age-risk group populations.

$N^{a,r}$	0-4	5-17	18-49	50-64	65 and older
Low risk	503894	1101232	2701594	778587	373036
High risk	38695	136609	624121	395585	344031

Supplementary Table 2: Houston age-risk group populations.

We define four baseline contact matrices, $\mathcal{H}, \mathcal{S}, \mathcal{W}$, and $\mathcal{O}$, to describe the contact frequency between age groups at home, at school, at work, and at other locations. These *baseline* matrices assume there is no difference in contacts among the low- and high-risk groups. Each row and column represents an age group, in the order of 0-4 years old, 5-17 years old, 18-49 years old, 50-64 years old, and 65 years old and above, with the row-column value corresponding to a "from-to" transmission contact:

$$\mathcal{H} = \begin{bmatrix} 0.5 & 0.9 & 2.0 & 0.1 & 0.0 \\ 0.2 & 1.7 & 1.9 & 0.2 & 0.0 \\ 0.2 & 0.9 & 1.7 & 0.2 & 0.0 \\ 0.2 & 0.7 & 1.2 & 1.0 & 0.1 \\ 0.1 & 0.7 & 1.0 & 0.3 & 0.6 \end{bmatrix} \qquad \mathcal{S} = \begin{bmatrix} 1.0 & 0.5 & 0.4 & 0.1 & 0.0 \\ 0.2 & 3.7 & 0.9 & 0.1 & 0.0 \\ 0.0 & 0.7 & 0.8 & 0.0 & 0.0 \\ 0.1 & 0.8 & 0.5 & 0.1 & 0.0 \\ 0.0 & 0.0 & 0.1 & 0.0 & 0.0 \end{bmatrix}$$

$$\mathcal{W} = \begin{bmatrix} 0.0 & 0.0 & 0.0 & 0.0 & 0.0 \\ 0.0 & 0.1 & 0.4 & 0.0 & 0.0 \\ 0.0 & 0.2 & 4.5 & 0.8 & 0.0 \\ 0.0 & 0.1 & 2.8 & 0.9 & 0.0 \\ 0.0 & 0.0 & 0.1 & 0.0 & 0.0 \end{bmatrix} \qquad \mathcal{O} = \begin{bmatrix} 0.7 & 0.7 & 1.8 & 0.6 & 0.3 \\ 0.2 & 2.6 & 2.1 & 0.4 & 0.2 \\ 0.1 & 0.7 & 3.3 & 0.6 & 0.2 \\ 0.1 & 0.3 & 2.2 & 1.1 & 0.4 \\ 0.0 & 0.2 & 1.3 & 0.8 & 0.6 \end{bmatrix}.$$

The contact matrices $\phi_{i,t}^{a',r',a,r}$ are calculated in the same way as Table S6 in [5], considering the effect of weekends, holidays, school closures, and physical distancing and cocooning of high-risk populations based on the risk stage. Stages correspond to distancing stages of different strictness, which govern the reduced number of daily contacts people make relative to baseline. In our model, this is reflected by a coefficient $\kappa_i, i \in \mathcal{I}$, where $\kappa_i = 0.75$ would reduce the expected number of contacts to 25% of the baseline value. For the age group of 65 years and older and for the high-risk group, we use reductions based on cocooning, which are represented by coefficients $c_i, i \in \mathcal{I}$:

$$\phi_{i,t}^{a',r',a,r} = \begin{cases} (1-\kappa_i)\left[(1-1_{\{\text{off day}\}})\cdot(1-1_{\{\text{school closure}\}})\cdot\mathcal{S}_{a',a}+ \right. & \text{if } a',a \in \{\text{0-4yr, 5-17yr, 18-49yr, 50-64yr}\}, \\ \quad \left.(1-1_{\{\text{off day}\}})\cdot\mathcal{W}_{a',a}+\mathcal{H}_{a',a}+\mathcal{O}_{a',a}\right] & \quad r',r \neq \text{high-risk} \\ (1-c_i)\left[(1-1_{\{\text{off day}\}})\cdot(1-1_{\{\text{school closure}\}})\cdot\mathcal{S}_{a',a}+ \right. & \\ \quad \left.(1-1_{\{\text{off day}\}})\cdot\mathcal{W}_{a',a}+\mathcal{H}_{a',a}+\mathcal{O}_{a',a}\right] & \text{otherwise.} \end{cases} \quad [4]$$

The indicator $1_{\{\text{off day}\}}$ takes value 1 if the day is a weekend or holiday and is otherwise 0, and a similar indicator accounts for school closures. When a high-risk group, along with those 65 years and older, is involved either on the "giving" or "receiving" end of a contact, Eq. [4] assumes reduced transmission via the cocooning coefficient, c_i.

The following are key dates during the pandemic in Texas, and some define time blocks, which we use in estimating time-varying transmission reduction factors and other key model parameters as we describe shortly:

- February 19, 2020: Seed date for simulation of Houston, assuming seeding by a single symptomatic individual age 18-49y. This corresponds to 14 days prior to the first detected COVID-19 case in Houston on March 4, 2020.
- February 28, 2020: Seed date for simulation of Austin, assuming seeding by a single symptomatic individual age 18-49y. This corresponds to 14 days prior to the first detected COVID-19 case in Austin on March 13, 2020.
- March 24, 2020: Austin's Stay Home-Work Safe Order is enacted at midnight [8]. On the same day, Harris County (Houston) issues Stay Home, Work Safe Order [9].
- May 1, 2020: The Governor of Texas relaxed physical distancing orders statewide [10].
- May 21, 2020: Just prior to Memorial Day Weekend.
- June 26, 2020: The Governor of Texas issued an executive order limiting service at bars and restaurants, and Travis County (which includes Austin) banned gatherings of more than 100 people [11, 12].
- July 17, 2020: Time point in hospitalization data suggesting a change in dynamics.
- August 9, 2020: The last day of observed data from the hospital system used in estimating changes in ICU dynamics.
- August 20, 2020: First day students returned to residence halls at the University of Texas at Austin.
- October 7, 2020: The last day of observed data used in estimating model parameters.

We assume that there are six time blocks denoted by $\mathcal{T}_j$ for $j \in \{1,2,3,4,5,6\}$ as defined in Supplementary Table 3. They guide fitting of transmission-reduction parameters, κ and c, and certain dynamics in use of the ICU and hospital duration, as detailed below.

Time Block	Start Date	End Date	Definition
$\mathcal{T}_1$	2/28/20 (Austin)	3/23/20	unmitigated transmission before first stay-home order
	2/19/20 (Houston)	3/23/20	
$\mathcal{T}_2$	3/24/20	5/20/20	effective period for first stay-home order
$\mathcal{T}_3$	5/21/20	6/25/20	relaxed period starting with Memorial Day weekend
$\mathcal{T}_4$	6/26/20	7/16/20	period of effective physical distancing
$\mathcal{T}_5$	7/17/20	8/19/20	period distinguished by changes in ICU dynamics
$\mathcal{T}_6$	8/20/20	10/7/20	period of effective physical distancing

Supplementary Table 3: The five time blocks, $\mathcal{T}_1$, $\mathcal{T}_2$, $\mathcal{T}_3$, $\mathcal{T}_4 \cup \mathcal{T}_5$, and $\mathcal{T}_6$ correspond to different rates of spread, as estimated using transmission-reduction factors κ and c. The fourth and fifth time blocks, $\mathcal{T}_4$ and $\mathcal{T}_5$, differ only in dynamics involving the ICU, both the admission probability and the sojourn time in the general ward prior to ICU admission.

We model the hospitalization dynamics, including proportions of hospitalized requiring the ICU and durations in the general ward and ICU, using data from a multi-facility hospital system serving the central Texas region, including Austin, Texas ("Hospital system data"). While we model differences based on five age groups, we assume the same hospital dynamics in different hospital systems after a patient is admitted across Austin and Houston due to similar medical standards. Conditional on being admitted to the hospital, we observe a decreasing trend in the probability a patient is admitted to the ICU throughout the time horizon, which holds for both direct admissions to the ICU and patients who are first admitted to the general ward. Among patients who enter the general ward and are then admitted to the ICU, their duration of stay in the general ward, determined by η_{ICU}, grows over time. For each time block, $\mathcal{T}_j$, we assume a constant $\eta_{ICU,j}$ and further assume a constant daily decrease, r_j, on both of the fractions, p_{IH} and $HICUR$:

$$p_{IH,t+1} = r_j p_{IH,t} \qquad \forall j \in \{1,2,3,4,5,6\}, t \in \mathcal{T}_j \qquad [5a]$$

$$HICUR_{t+1} = r_j HICUR_t \qquad \forall j \in \{1,2,3,4,5,6\}, t \in \mathcal{T}_j, \qquad [5b]$$

along with a similar decrement across boundaries of the blocks. We use duration times for each time block from the hospital system data to estimate $\eta^a_{ICU,j}$ and fit r_j, with the estimated parameters in Supplementary Table 4.

	age group	$\mathcal{T}_1$	$\mathcal{T}_2$	$\mathcal{T}_3$	$\mathcal{T}_4$	$\mathcal{T}_5 \cup \mathcal{T}_6$
$\eta^a_{ICU,j}$	0-4 yr	0.5882	0.5882	0.3885	0.2640	0.2589
	5-17 yr	0.5882	0.5882	0.3885	0.2640	0.2589
	18-49 yr	0.5882	0.5882	0.3885	0.2640	0.2589
	50-64 yr	0.6273	0.6273	0.4143	0.2815	0.2761
	$\geq$ 65 yr	0.6478	0.6478	0.4278	0.2907	0.2851
r_j		0.9973	0.9973	0.9932	0.9921	1

Supplementary Table 4: Estimates of ICU admission probability parameters, η_{ICU}, p_{IH}, and $HICUR$; see Supplementary Figure 1 and accompanying parameter definitions. For each age group, a, and each time block, j, we specify η_{ICU}, and we give the daily decrement factor, r_j, used in Eq. [5].

Using the hospital system data, and consistent with the transition diagram in Supplementary Figure 1, we define the ICU duration for a patient as the time between their admission to the ICU and their discharge from the hospital. The reality is more complex as ICU patients typically return to the general ward prior to discharge from the hospital, and iterations between the two units, driven by a patient's health status, can also occur. Therefore, the reported duration in the ICU leads to over estimating ICU utilization and under-estimating that of the general ward. To handle this in our model, we introduce two constant parameters, α_{ICU} and α_H, to better estimate durations in the ICU and general ward and better represent their respective utilization:

$$\gamma_H = (1 - \alpha_H)\gamma^0_H$$
$$\gamma_{ICU} = (1 + \alpha_{ICU})\gamma^0_{ICU}$$
$$\mu = (1 + \alpha_{ICU})\mu^0,$$

where γ^0_H, γ^0_{ICU}, and μ^0 are obtained from the hospital system data, with each row corresponding to an age group in ascending order:

$$\gamma^0_H = \begin{bmatrix} 0.2399 \\ 0.2399 \\ 0.2399 \\ 0.2222 \\ 0.2124 \end{bmatrix}, \ \gamma^0_{ICU} = \begin{bmatrix} 0.0700 \\ 0.0700 \\ 0.0700 \\ 0.0575 \\ 0.0518 \end{bmatrix}, \ \mu^0 = \begin{bmatrix} 0.0749 \\ 0.0749 \\ 0.0749 \\ 0.0766 \\ 0.0799 \end{bmatrix},$$

with units of day^{-1}.

The bulk of the epidemiological and hospitalization parameters are specified above or are detailed in Supplementary Tables 10 and 11, with the latter obtained from the literature or information collected from local healthcare agencies. The time blocks are specified in Supplementary Table 3. Given these, we estimate 14 parameters, but with 7 degrees of freedom, as we detail below. We perform the fit of the deterministic SEIR model in Eqs. [1] using: (i) daily COVID-19 admissions, denoted H_t and available for Austin only; (ii) a daily COVID census in the general ward, IH_t; and (iii) a daily COVID census in the ICU, ICU_t, all on day t. The fit is performed separately for the Austin MSA and Houston MSA. By minimizing a weighted sum of least-square errors, we estimate $\hat{\kappa}_j$ and $\hat{c}_j$, $j = 1, 2, \ldots, 6$, α_H, and α_{ICU}, using SciPy/Python [13] via `scipy.optimize.least_squares`. We use the "hat" notation on κ and c to distinguish, for example, $\hat{\kappa}_j$ for *time block* j (see Supplementary Table 3) from κ_i, which corresponds to *stage* i per Eq. [4], and show the mapping shortly.

We minimize

$$\sum_t (IH_t - \widehat{IH}_t)^2 + w_{ICU}^2 \sum_t (ICU_t - \widehat{ICU}_t)^2 + w_H^2 \sum_t (H_t - \widehat{H}_t)^2,$$

where $\widehat{IH}_t$, $\widehat{ICU}_t$, and $\widehat{H}_t$ denote the estimated IH_t, ICU_t, and H_t obtained through Eqs. [1]; w_{ICU} and w_H are scaling constants; and the sum is over $t \in \mathcal{T}_1 \cup \cdots \cup \mathcal{T}_6$. We assume $w_{ICU} = 1.50$ and $w_H = 7.58$, as those values approximate magnitudes relative to that of the general ward. To obtain a parsimonious model, we use $\hat{c}_1 = 0$, $\hat{c}_2 = \hat{c}_3 = \hat{\kappa}_2$, $\hat{c}_4 = \hat{c}_5 = \hat{\kappa}_4 = \hat{\kappa}_5$ and $\hat{c}_6 = \hat{\kappa}_6$, which reduces the number of estimated parameters from 14 to 7. The rationale is that there was effectively no cocooning during the initial time block $\mathcal{T}_1$, and thus we set $\hat{c}_1 = 0$. Because physical distancing was stricter over time block $\mathcal{T}_2$ than time block $\mathcal{T}_3$, and the cocooning effectiveness parameters are expected to be at least that of the distancing parameters, we use $\hat{c}_2 = \hat{c}_3 = \hat{\kappa}_2$. Because of behavioral changes over time, including increased use of face-masks, when we perform the fit we observe greater reduction in transmission in time blocks 4 and 5 than we do in block 2, and so we use $\hat{c}_4 = \hat{\kappa}_4$. Finally, while hospitalization parameters differ over time blocks $\mathcal{T}_4$ and $\mathcal{T}_5$, rates of transmission do not appear to differ significantly, and so we consider $\hat{c}_4 = \hat{c}_5 = \hat{\kappa}_4 = \hat{\kappa}_5$.

We use the trust region reflective algorithm (`trf`) in `scipy.optimize.least_squares`, with lower and upper bounds on each parameter of 0 and 1, respectively. The algorithm obtains locally optimal values of the parameters, the quality of which has been validated by comparing projections with the observed data. All the remaining parameters are set to their default values (see above and Supplementary Tables 10 and 11). The fitted values for $\hat{\kappa}_j$ and $\hat{c}_j$ and α_H and α_{ICU} are given in Supplementary Table 5 for both Austin and Houston.

	Austin		Houston	
j	$\hat{\kappa}_j$	$\hat{c}_j$	$\hat{\kappa}_j$	$\hat{c}_j$
1	0.0613	0.0000	0.1168	0.0000
2	0.7436	0.7436	0.7304	0.7304
3	0.6026	0.7436	0.5643	0.7304
4	0.7815	0.7815	0.7553	0.7553
5	0.7815	0.7815	0.7553	0.7553
6	0.7544	0.7544	0.7118	0.7118
α_H	0.2862		0.4858	
α_{ICU}	0.5621		0.4876	

Supplementary Table 5: Fitted transmission reduction parameters, $\hat{\kappa}_j$, and cocooning effectiveness parameters, $\hat{c}_j$, for each time block $\mathcal{T}_j$, along with estimated hospitalization duration adjustment parameters, α_H and α_{ICU}, for both Austin and Houston.

The physical distancing parameters for each stage are mapped to κ_i and c_i for $i \in \mathcal{I}$ based on the historical

implementation of the policy. We set:

$$\kappa_1 = \hat{\kappa}_4 \qquad c_1 = \hat{\kappa}_4 \tag{6a}$$

$$\kappa_2 = \frac{\hat{\kappa}_4 + \hat{\kappa}_3}{2} \qquad c_2 = \hat{\kappa}_2 \tag{6b}$$

$$\kappa_3 = \hat{\kappa}_3 \qquad c_3 = \hat{\kappa}_2 \tag{6c}$$

$$\kappa_4 = \hat{\kappa}_3 - \frac{\hat{\kappa}_4 - \hat{\kappa}_3}{2} \qquad c_4 = 1 - 1.25 \cdot (1 - c_2). \tag{6d}$$

In Eq. [6a] we set κ_1 (red) to the strictest observed level of transmission reduction, June 26–August 19, 2020. In Eq. [6c] we set κ_3 (yellow) to the relaxed period of relatively high transmission, roughly from Memorial Day weekend through June 25, 2020. Eq. [6b] forms κ_2 (orange) as the average between these two levels of reduction. The most relaxed stage of transmission reduction we consider (blue), uses the same increment between the red and orange stages and between orange and yellow in Eq. [6d]. We do not separately estimate cocooning, but assume it matches the strictest level of reduction in the low-risk population when in the red stage, that observed during the stay-home period after March 24th for the orange and yellow stages, and then in the blue stage relaxes by a 25% increment, as shown in Eq. [6d], so that a reduction of 0.75 would drop to 0.6875.

Algorithm 1: Pseudo-code for the sampling procedure

Initialize $\Omega = \emptyset$
while $|\Omega| < 300$ **do**
  for $j = 1, \ldots, 6$ **do**
    if $j == 1$ **then**
      Generate a scenario (sample path) ω over time block $\mathcal{T}_1$
    else
      Continue simulating scenario ω over time block $\mathcal{T}_j$
    Compute $R^2 = 1 - \frac{\sum_{t \in \mathcal{T}_1 \cup \cdots \cup \mathcal{T}_j} (IH_t - \widehat{IH}_t)^2}{\sum_{t \in \mathcal{T}_1 \cup \cdots \cup \mathcal{T}_j} (IH_t - \overline{IH})^2}$
    if $R^2 < 0.85$ **then**
      break and initiate next ω and $j = 1$
  $\Omega \leftarrow \Omega \cup \{\omega\}$

Supplementary Method 3. Selection of Scenarios

After fitting the parameters via least-squares minimization using the procedure just described, we simulate a set of scenarios, Ω. There are macro stochastics, involving modeling σ, ω_A, γ_A, and γ_Y as random variables; see Supplementary Table 10. And, micro stochastics govern a binomially distributed number of transitions between compartments. Inevitably, some stochastic sample paths will yields hospitalizations that diverge from observed data, including sample paths in which spread quickly terminates after initializing with a single infectious person.

Here we describe how we select scenarios, sampled from the marginal distributions of σ, ω_A, γ_A, and γ_Y, and from the micro-stochastics process, in order to generate a set of 300 scenarios, indexed by $\omega \in \Omega$ and used in Eqs. [1]. Because the projections at earlier time points guide the trigger optimization model at later time points, the goal is to start the latter with scenarios, $\omega \in \Omega$, that are consistent with observed hospitalizations up to that point in time. In

order to evaluate the quality of scenario ω in this sense, we use

$$R^2 = 1 - \frac{\sum_t (IH_t - \widehat{IH}_t)^2}{\sum_t (IH_t - \overline{IH})^2},$$

where $\overline{IH}$ is the mean of the IH_t values. Algorithm 1 summarizes the sampling procedure to generate $|\Omega| = 300$ scenarios.

Algorithm 1 uses the six time blocks $\mathcal{T}_j$, $j = 1, 2, 3, 4, 5, 6$, given in Supplementary Table 3. We start with simulating a sample path ω during the first time block $\mathcal{T}_1$ (see line 4–5), and compute the corresponding R^2 value at the end of $\mathcal{T}_1$. If the scenario, ω, has the desired quality over $\mathcal{T}_1$, i.e., $R^2 \geq 0.85$, we continue simulating the path ω. At the end of each time block, we compute the corresponding R^2 value as shown in line 8, and decide whether the path is kept or discarded, as shown in lines 9–10. Here, the threshold value 0.85 of R^2 is a tuning parameter, which we return to below. Once Algorithm 1 provides 300 scenarios at the end of time block $\mathcal{T}_6$, we continue simulating them for use in the stochastic optimization model [3] over the rest of the time horizon.

Supplementary Figure 2 shows histograms of the resulting marginal distributions from Algorithm 1 for σ, ω_A, γ_A, and γ_Y. The scatter plots of Supplementary Figure 3 again use the scenarios, Ω, obtained by Algorithm 1 to give insight to bivariate dependencies. We generate random variables from the independent nominal triangular distributions given in Supplementary Table 10, and Algorithm 1 "accepts" those that are consistent with observed hospitalizations. The resulting marginal distributions can differ from the nominal distribution; see the distribution of ω_A in Supplementary Figure 2. In addition, even though we sample independently from the nominal distributions, Algorithm 1 can induce dependence; see the strong negative correlation between ω_A and $1/\gamma_Y$ in Supplementary Figure 3.

In Algorithm 1, we use a threshold of 0.85 for R^2, through each time block, to decide whether the generated path satisfies the desired quality through October 7, 2020. Of course, different thresholds are possible. To understand how sensitive the optimized policies are for different threshold values, we also obtain optimized policies using the scenarios generated with two more thresholds for R^2, 0.65 and 0.75, for Austin. Supplementary Figure 4 illustrates the projections of new daily hospitalizations and ICU hospitalizations. Analogs of the projections for those thresholds are also visualized in Fig. 1 in the main text for a threshold of 0.85. There are not dramatic differences across these three thresholds for R^2. The optimized policies are the same for thresholds of 0.65 and 0.75, and we observe a slightly smaller trigger (120 versus 130) for the red stage for an R^2 threshold of 0.85. As the threshold values shrink from 0.85 to 0.75 to 0.65: the expected number of days in the most stringent red-stage lock-down are 7.6, 5.4, and 5.8; the 95th percentiles of peak hospitalizations are 993, 1033, and 1028 (capacity is 1500); and the 95th percentiles of peak ICU demand are 317, 327, and 347 (capacity is 331). Here, the trigger policy is optimized for ICU capacity, and we observe occasional non-monotonic behavior due to statistical noise. That said, these results, along with Supplementary Figure 4, suggest that our results are not overly sensitive to our choice of 0.85 for the threshold.

For the purpose of visualizing results we use spaghetti plots that detail 300 sample paths, and we also use a single "representative" path. A simple least-squares selection of a path from the collection of 300 paths arguably does *not* yield a representative path. Because the timing of peaks in paths differs, the simple least-squares path is "flatter" through the cloud than most paths. Here we describe how we select a representative path from the collection of 300.

We define a selection criterion based on metrics of interest, such as the total number of people hospitalized in the general ward over the duration, the peak number of people in the hospital, and the time of that peak. We let $\widehat{IH}_{t,\omega}$, $\widehat{ICU}_{t,\omega}$, and $\widehat{H}_{t,\omega}$ denote the estimates of IH_t, ICU_t, and H_t for each sample path, $\omega \in \Omega$, and we first obtain the squared deviations from the observed data, defining $Z_{\omega,obs}$ as follows:

$$Z_{\omega,obs} = \sum_t (\widehat{IH}_{t,\omega} - IH_t)^2 + w_{ICU}^2 \sum_t (\widehat{ICU}_{t,\omega} - ICU_t)^2 + w_H^2 \sum_t (\widehat{H}_{t,\omega} - H_t)^2,$$

where w_{IH} and w_{ICU} are scaling constants. Here, t ranges over all days up to October 7, 2020, for which we have observed data. We then define metrics to represent the statistical properties of the scenarios as in Supplementary Table 6.

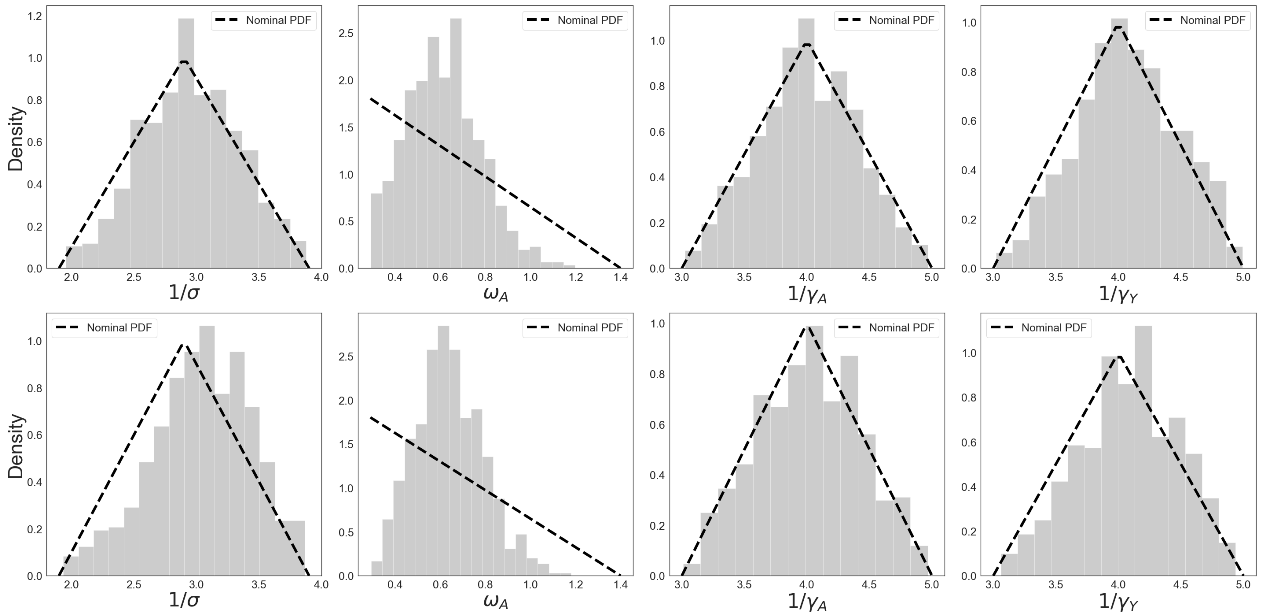

Supplementary Figure 2: Histogram of the random variables σ, ω_A, γ_A, and γ_Y for the scenario set Ω obtained by Algorithm 1. The first and second row represent the set of scenarios for Austin and Houston, respectively. Rather than showing the rates, we show $1/\sigma$, $1/\gamma_A$, and $1/\gamma_Y$, which correspond to durations in days. We sample from the marginal distributions of each of the four parameters, and Algorithm 1 accepts sample paths consistent with hospitalizations, while also sampling a binomial number of transitions between compartments in the SEIR-style model. While there are modest deviations from the nominal triangular distributions for durations ($1/\sigma$, $1/\gamma_A$, and $1/\gamma_Y$), the distribution of the relative infectiousness of an asymptomatic individual (ω_A) deviates significantly from its nominal distribution. Here, σ is the rate at which exposed individuals become infectious; ω_A is the infectiousness of asymptomatic relative to symptomatic individuals; and γ_A and γ_Y are recovery rates from the asymptomatic and symptomatic compartments.

Metric	Definition	Standardized Metric
$IH_{\omega,tot} = \sum_{t=1}^{T} IH_{t,\omega}$	Total number of patient-days	$Z_{\omega,IH_{tot}} = (IH_{\omega,tot} - \hat{\mu}_{IH_{tot}})/\hat{\sigma}_{IH_{tot}}$
$IH_{\omega,max} = \max_t IH_{t,\omega}$	Peak hospitalization	$Z_{\omega,IH_{max}} = (IH_{\omega,max} - \hat{\mu}_{IH_{max}})/\hat{\sigma}_{IH_{max}}$
$IH_{\omega,med} = \text{median}_t IH_{t,\omega}$	Median hospitalization	$Z_{\omega,IH_{med}} = (IH_{\omega,med} - \hat{\mu}_{IH_{med}})/\hat{\sigma}_{IH_{med}}$
$t_{\omega,IH_{max}} = \arg\max_t IH_{t,\omega}$	Timing of hospitalization peak	$Z_{t_{\omega,IH_{max}}} = (t_{\omega,IH_{max}} - \hat{\mu}_{t_{IH_{max}}})/\hat{\sigma}_{t_{IH_{max}}}$

Supplementary Table 6: Metrics for hospitalized patients in the general ward under each sample path ω. Note that $\hat{\mu}$ and $\hat{\sigma}$ represent the sample mean and sample standard deviation for each metric, across the $|\Omega| = 300$ sample paths.

Analogs of the standardized metrics for those hospitalized in the general ward (IH) from Supplementary Table 6 are also computed for those hospitalized in the ICU (ICU), and new daily hospital admissions (H) to form a total of 12 such metrics. We select the representative scenario ω' such that

$$\omega' \in \arg\min_{\omega\in\Omega} \left(\frac{w_{std}}{12} Z_{\omega,std} + w_{obs} Z_{\omega,obs} \right) \qquad [7]$$

where

$$\begin{aligned} Z_{\omega,std} = {} & Z^2_{\omega,IH_{tot}} + Z^2_{\omega,IH_{max}} + Z^2_{\omega,IH_{med}} + Z^2_{\omega,t_{IH_{max}}} \\ & + Z^2_{\omega,ICU_{tot}} + Z^2_{\omega,ICU_{max}} + Z^2_{\omega,ICU_{med}} + Z^2_{\omega,t_{ICU_{max}}} \\ & + Z^2_{\omega,H_{tot}} + Z^2_{\omega,H_{max}} + Z^2_{\omega,H_{med}} + Z^2_{\omega,t_{H_{max}}}, \end{aligned}$$

where w_{std} and w_{obs} are positive weights that sum to one. This method is used to select the representative paths

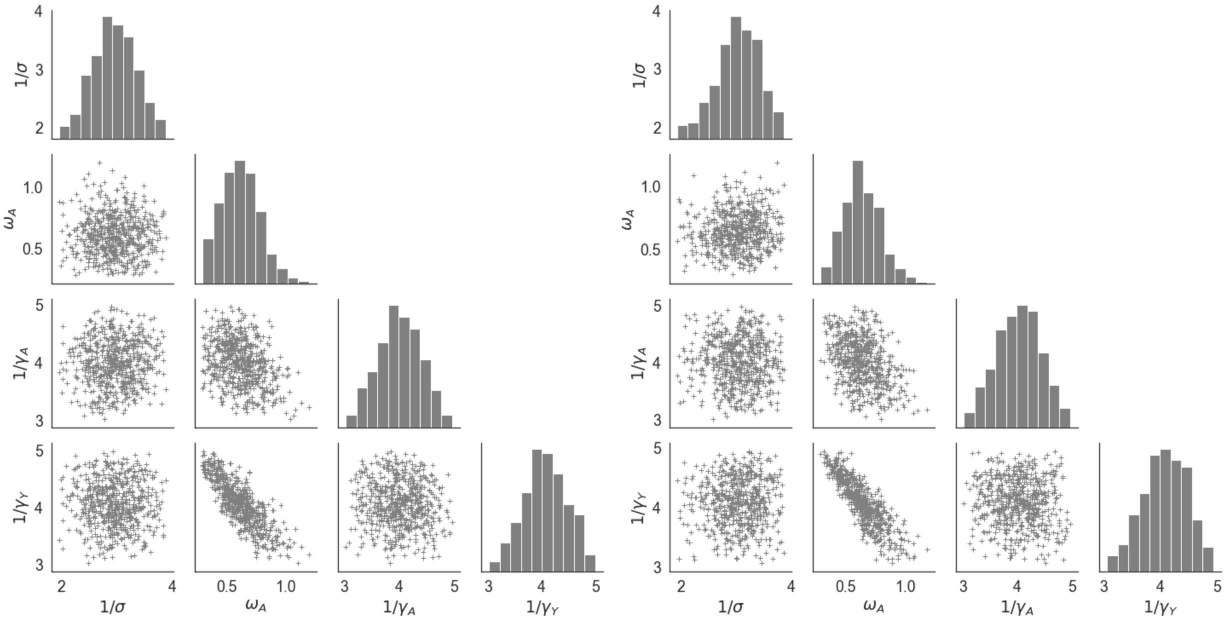

Supplementary Figure 3: Scatter plot of the random variables $1/\sigma$, ω_A, $1/\gamma_A$, and $1/\gamma_Y$ for the scenario set Ω selected by Algorithm 1. The left and right scatter plots represent the set of scenarios, Ω, for Austin and Houston, respectively. We sample *independently* from the marginal distributions of each of the four parameters, and the algorithm accepts sample paths consistent with hospitalizations. This can induce dependencies as shown, for example, between $1/\gamma_Y$ and ω_A. Here, σ is the rate at which exposed individuals become infectious; ω_A is the infectiousness of asymptomatic relative to symptomatic individuals; and γ_A and γ_Y are recovery rates from the asymptomatic and symptomatic compartments.

plotted in Fig. 1 in the main text, and Supplementary Figures 4-5 and Supplementary Figures 8-9. Moreover, when we have paired plots of total hospitalizations, or ICU hospitalizations, along with daily admissions (see Fig. 1 in the main text and Supplementary Figures 4-5) then the cyan and black curves correspond to the same scenario.

Two key factors play a role the Monte Carlo acceptance-rejection sampling procedure of Algorithm 1. One is the nature of the stochastics, i.e., macro-stochastic and micro-stochastics. The second is the initial "burn-in" period of February 28–March 23, 2020 (for Austin), which plays an important role, in part because we start with a single infectious individual on February 28th. In our analysis, we generate two sets of scenarios that are similar to "training" and "testing" data used in statistics and machine learning. We use 300 scenarios for each set, and for Austin this required generating 918,220 total scenarios; i.e., the acceptance rate is about 0.07%. Of those scenarios, all but 33,964 were rejected during the initial 25-day period; i.e., conditional on not being rejected in the burn-in period the acceptance rate jumps by a factor of about 25 to about 1.8%. In order to assess the effect of micro- and macro-stochastics on our Monte Carlo procedure, we restrict attention to scenarios that have not been rejected during the burn-in period. First, among the scenarios that are ultimately accepted (see Supplementary Figure 2) we select the centroid in order to eliminate macro-stochatics. Conditioning on not being rejected during the initial time period, in order to accept 600 scenarios we must generate 17,030 scenarios, meaning the acceptance rate jumps by a factor of about two. Next, we eliminate micro-stochastics by modeling the transitions from one compartment to another based on the expected value, i.e., the deterministic rate associated with the binomial random variables, rather than using samples from the binomial random variables. Again conditioning on not being rejected in the initial 25-day period, the acceptance rate jumps to about 12%.

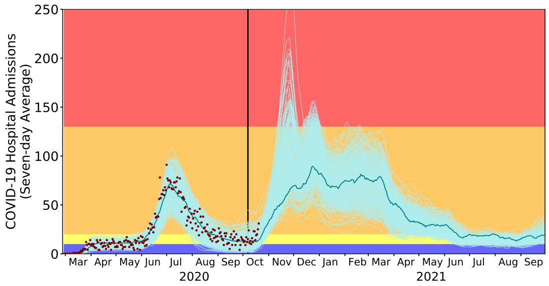

(a) Daily Hospital Admissions: Austin ($R^2 \geq 0.65$)

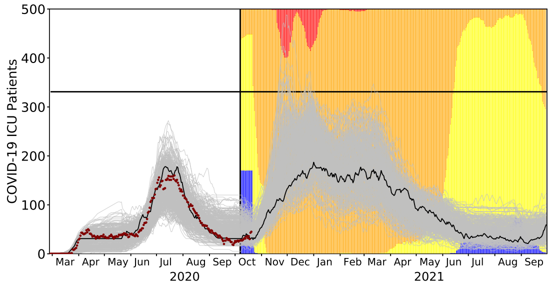

(b) ICU Hospitalizations: Austin ($R^2 \geq 0.65$)

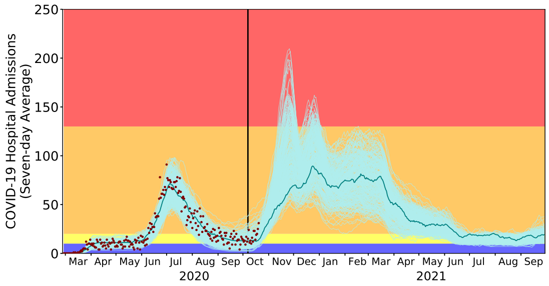

(c) Daily Hospital Admissions: Austin ($R^2 \geq 0.75$)

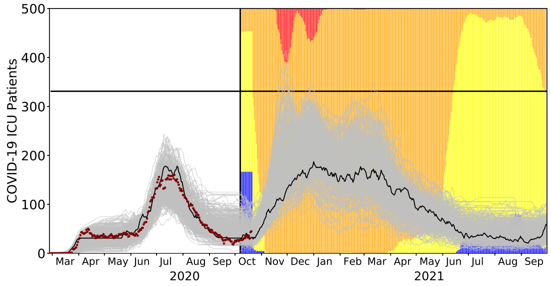

(d) ICU Hospitalizations: Austin ($R^2 \geq 0.75$)

Supplementary Figure 4: COVID-19 healthcare projections for Austin under the optimized staging policy, from October 7, 2020 through September 30, 2021. Each row represents a projection with a different threshold value for R^2 in Algorithm 1, and are analogs of Fig. 1 in the main text, which uses $R^2 \geq 0.85$. The strategies were derived to minimize the expected days in costly alert stages while respecting intensive care unit (ICU) capacity. The light curves indicate 300 stochastic simulations, the single solid curves are representative central projections, the red points correspond to the reported COVID-19 admissions and ICU census for all Austin area hospitals through October 20, 2020, and the vertical black line indicates the start of the projection period. (a,c) The optimized policy triggers changes in the COVID-19 alert level when the seven-day moving average of daily COVID-19 hospital admissions crosses optimized thresholds, as indicated by the colored horizontal bands for the red, orange, yellow, and blue alert stages. The alert stage shifts when the admissions indicator surpasses or recedes below the corresponding threshold. (b,d) The policy provides a 95% guarantee that the number of COVID-19 ICU patients does not exceed the estimated local capacity of 331 patients (black horizontal line). The background colors represent the proportion of the simulated scenarios in each alert stage on each day. Optimized policies that keep us within ICU capacity are identical under thresholds 0.65 and 0.75, and there is a modest change in the red stage's trigger (represented by the red horizontal band) for a threshold of 0.85.

Supplementary Discussion 1. Additional Analysis

Test Settings

In Section 2 of the main text we compare four alternative policies to our optimized four-stage trigger policy. First, we test a benchmark which is again optimized and differs from the four-stage policy only in that it has access to just two stages of mitigation, red and yellow. In this case, we require a single threshold to identify when to toggle between the two stages. In our second benchmark, we again optimize in the same way as the four-stage policy, but we do so using a 0.95-level probabilistic constraint to respect *total* hospital capacity rather than ICU capacity.

We assess two additional benchmarks. Instead of triggering based on the seven-day moving average of daily hospital admissions, we use the seven-day moving average of (i) daily census in the ICU and (ii) the estimated prevalence of infectious individuals. Alternative (i) is currently used in France [14], and in this case we use respective trigger thresholds for the red, orange, and yellow stages based on 60%, 30%, and 0% of the ICU capacity. Alternative (ii) was proposed by Harvard Global Health Institute [15], and stages red, orange, yellow, and blue correspond to more than 25, 10 to 25, 1 to 10, and less than 1 daily confirmed infectious case(s) per 100 000 people. Those thresholds

are adjusted with the population of Austin MSA (2.2 million) and Houston MSA (7.1 million), and use a 1-in-10 ratio between the confirmed infectious cases and total estimated infectious cases.

Supplementary Table 7 details parameters used in the ACS analysis in the next section for the Austin MSA, involving weighted combinations of transmission reduction between the orange and yellow stages. The stages differ in the physical distancing coefficient, κ, but all use the same cocooning coefficient, c, as shown in the table.

Transmission Reduction		κ	c
orange	yellow		
75%	25%	0.6697	0.7436
50%	50%	0.6473	0.7436
25%	75%	0.6250	0.7436
0%	100%	0.6026	0.7436

Supplementary Table 7: The two left-most columns indicate the relative weight on transmission reduction factors for the orange and yellow stages. So, the last row corresponds to yellow, while the 50-50% row corresponds to equal weight on the orange and yellow values. The two right-most columns show the resulting transmission reduction coefficients for the low-risk population (κ) and the high-risk population (c) for Austin.

We assume the ACS can be set up within two days; i.e., the ACS will be ready to use two days after the trigger threshold is reached. In reality, an ACS is built in phases with staffing being the final phase that allows the facility to function. The trigger threshold to operationalize and size the ACS are both selected by a grid search with the grid step sizes of 10 and 100, respectively

Optimal Triggers: Houston

We present results for Houston in a parallel format as for Austin in Section 2 of the main text. Houston's nine-county MSA has a population of 7.1 million. We use the estimated transmission rates from February 19–October 7, 2020 as given in Supplementary Tables 3 and 5. Detailed results for our proposed policy and the four benchmarks are shown in Supplementary Table 8 and we visualize the disease dynamics associated with the optimal triggers by new daily hospitalizations and the ICU daily census in Supplementary Figure 5, parallel to Table 1 and Fig. 1 in the main text. We also visualize the benchmark comparison results in Supplementary Figure 6, like in Fig. 2 in the main text. Qualitatively the results are similar to those for Austin except that for Houston the *Percent ICU* policy is reliable, albeit at a larger socioeconomic cost than the *Optimal* policy.

Construction of an Alternate Care Site

The success of trigger-based policies for ICU and hospital capacity depends on public adherence, which is not guaranteed. In case of failure, Austin applied this framework to determine both a threshold for launching an alternate care site (ACS) and the required capacity. The city planned to transfer low acuity COVID-19 patients to the ACS when cases exceed the city-wide estimated 1500 patient capacity. We consider four scenarios for non-adherence, each assuming a constant transmission rate between those estimated for Austin under restrictions of the orange and yellow stages. The lowest adherence scenario assumes the transmission rate estimated in Austin from Memorial Day (late-May) through late-June of 2020, after *Opening Up America Again* was promulgated and before the city and state took measures to curb the early summer surges, including strict face-mask requirements. In this situation, the ACS should be stood up when the seven-day rolling average of COVID-19 hospital admissions reaches 110; the recommended trigger increases to 220 under the highest adherence scenario (Supplementary Table 9 and Supplementary Figure 7). Failing to construct an ACS would lead to a median of 30519 [90% PI: 11834-62450] and 0.0 [90% PI: 0-255] patient-days of unmet demand, under these two scenarios, respectively. We would expect demand for ACS beds to last up to two months, and to decrease with higher levels of adherence.

Sensitivity Analysis

We report additional results on the sensitivity associated with public compliance with recommended physical distancing, or lack thereof. Supplementary Figures 8a and 8b show results assuming that after October 7th the transmission reduction level stays at that of the yellow stage. The ICU hospitalizations greatly exceed the ICU capacity in all 300 scenarios for both Austin and Houston. Median peak ICU hospitalizations are 810 for Austin (capacity 331)

		Policies				
		Optimal (ICU capacity)	Optimal two-stage (ICU capacity)	Optimal hospital (overall capacity)	Percent ICU (France)	Incidence (Harvard)
Indicator data		COVID-19 hospital admissions (seven-day average)			percent ICU beds occupied by COVID-19	new cases per 100 000 (seven-day average)
Thresholds	blue (new normal)	<30	—	<30	—	<1
	yellow (moderate risk)	30-90	<380	30-160	<30%	1-10
	orange (high risk)	90-500	—	160-560	30%-60%	10-25
	red (very high risk)	>500	>380	>560	>60%	>25
Median days in red stage [90% PI]		0 [0-0]	15 [14-30]	0 [0-0]	16 [0-23]	0 [0-14]
Probability ICU demand exceeds capacity		2.3%	2.3%	53.7%	0.0%	0.0%
Median peak ICU demand [patients]		780	901	1010	658	303
95th percentile of peak ICU demand		977	984	1187	740	395
Median unserved ICU demand (patient-days) [90% PI]		0 [0-0]	0 [0-0]	15 [0-3915]	0 [0-0]	0 [0-0]
99th percentile of unserved ICU demand (patient-days)		540	24	6843	0	0

Supplementary Table 8: Performance comparison across five COVID-19 staging policies for Houston. From left to right, the three optimal policies are derived to prevent overwhelming ICU demand using either a four-level or two-level alert system or to prevent overwhelming inpatient demand using a four-level system. As benchmarks, we evaluate policies implemented in France [14] and proposed as gating criteria for relaxing measures and opening schools in [15]. For Houston, the orange and red thresholds for the *Percent ICU* policy translate to 300 and 600 COVID-19 ICU cases, respectively; the yellow, orange, and red thresholds for the *Incidence* policy translate to 710, 7100, and 17750 new cases, respectively, assuming that one in ten cases is reported. We implemented each policy in our stochastic SEIR model fit to hospitalization data for the Houston, Texas MSA assuming the reported COVID-19 ICU and inpatient capacities of 1000 and 4500 beds, respectively. Outcomes are based on 300 stochastic simulations of COVID-19 transmission and healthcare burden from October 7, 2020 through September 30, 2021 under each policy.

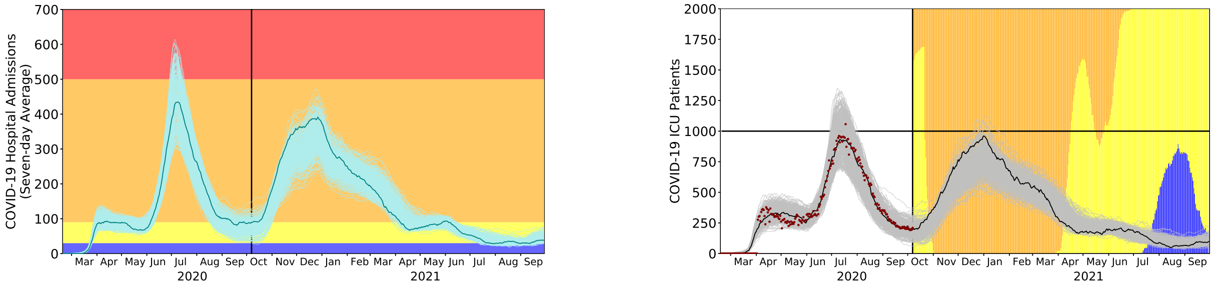

(a) Daily Hospital Admissions: Houston

(b) ICU Hospitalizations: Houston

Supplementary Figure 5: COVID-19 healthcare projections for Houston under the optimized staging policy, from October 7, 2020 (marked by the vertical line) through September 30, 2021. The trigger strategy was derived to minimize the expected days in costly alert stages while respecting intensive care unit (ICU) capacity. In both plots, the light curves indicate 300 stochastic simulations, the single solid curve is a representative *central* projection, and the red points correspond to the ICU census for all Houston MSA hospitals through October 20, 2020. (a) The optimized policy triggers changes in the COVID-19 alert level when the seven-day moving average of daily COVID-19 hospital admissions crosses set thresholds, as indicated by the colored horizontal bands for the red, orange, yellow, and blue alert stages. The alert stage shifts when the admissions indicator surpasses or recedes below the corresponding threshold. (b) The policy provides a 95% guarantee that the number of COVID-19 ICU patients does not exceed the estimated local capacity of 1000 patients (black horizontal line). The background colors represent the proportion of the simulated scenarios in each alert stage on each day.

and 2574 for Houston (capacity 1000) with corresponding 95th percentiles of 1094 and 2920. For Austin and Houston we estimate a median 20366 and 70274 unserved patient-days in the ICU, with respective 90% prediction intervals of [13505-30575] and [55077-84364].

Supplementary Figures 8c and 8d show results that suppose optimized triggers from Table 1 in the main text are carried out but the high-risk population is not cocooned, and is instead subject to transmission rates in the low-risk

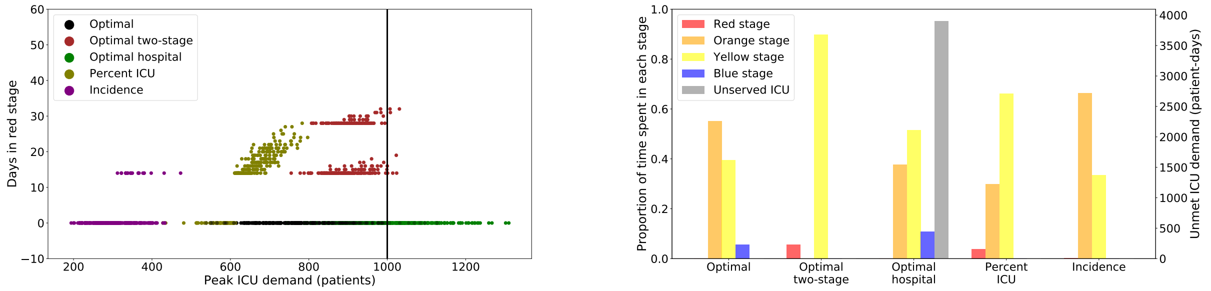

(a) Days in lock-down (red) versus peak ICU demand

(b) Proportion of time per stage and unserved ICU patient-days (95th percentile)

Supplementary Figure 6: Projected intensive care unit (ICU) surges and days under lock-down for the optimized strategy versus four alternative policies. *Optimal* is the recommended strategy. *Optimal two-stage* is optimized to respect ICU capacity under a two-level alert system, and *Optimal hospital* respects total hospital capacity under a four-level alert system. *Percent ICU* is based on France's lock-down policy [14] and *Incidence* is based on reopening criteria proposed by Harvard [15]. (a) The maximum daily number of COVID-19 patients in ICUs versus the number of days under the most restrictive alert level of red. Each point represents the result of a single stochastic simulation under one of the five policies (indicated by color). The plot includes 300 points per policy; the vertical gray line indicates the estimated COVID-19 ICU capacity of 1000 patients for the Houston area. The *Optimal* policy is designed to minimize the use of the strictest stages of mitigation while having 95% of the peak-demand values within ICU capacity. (b) The expected proportion of days spent in each stage, colored in the same manner as Supplementary Figure 5 and the 95th percentile of ICU shortage measured in patient-days above capacity, in gray with values indicated on the right y-axis.

Scenario		Launch	Target	Probability	95th Percentile	
Adherence	Transmission rate	trigger	capacity	launched	Days used	Peak demand
moderate-high	75%-25%	220	100	0.09	41	55
moderate	50%-50%	200	700	0.64	61	640
moderate-low	25%-75%	210	1400	0.97	63	1292
low	0%-100%	110	2000	1.00	65	1906

Supplementary Table 9: Optimized ACS policies under four adherence scenarios for the Austin area. The scenarios assume that the public do not fully comply with enacted alert levels, resulting in a constant transmission rate lying somewhere between those expected under the orange and yellow stages. The percents in the second column indicate the relative weighting of the orange and yellow rates; for example, the moderate-high rate is obtained via a 75-25 weighting of the two rates, respectively. The ACS policies track the moving seven-day average of COVID-19 hospital admissions and assume that the ACS will be ready to accept patients two days after triggering. The *launch trigger* is the derived threshold for opening the ACS; the *target capacity* is the estimated total ACS beds needed, based on the criteria that 95% of projections remain under ACS capacity; *probability launched* is the fraction of simulations in which admissions exceed the launch trigger; the 95th percentiles of *days used* and *peak demand* provide upper bounds on the projected demand. Coupled optimization of the the trigger and capacity in discrete steps of 100 beds and 10 daily admissions, respectively, along with the resulting slack between peak demand and target capacity can produce small non-monotonicities in optimal triggers, as seen in the middle two rows. We assume the ACS remains open for 100 days once triggered, which is more than sufficient to cover the estimated 95th percentile of days required (penultimate column).

population. In this case, we violate ICU capacity in 4.3% of the 300 out-of-sample scenarios in Austin and 97.3% of the scenarios in Houston. The 95th percentile of peak ICU hospitalizations grows to 329 for Austin (capacity 331) and 1231 for Houston (capacity 1000). After October 7, 2020, for Austin, we estimate a median 0 unserved patient-days, and for Houston, the corresponding estimate is 1626, with respective 90% prediction intervals of [0-0] and [76-4014].

In Supplementary Figure 9 we show results of tests for four situations in which the public complies with the optimal policy up to a certain date and after that date, compliance fails and transmission matches that of the yellow stage. The figure shows results when such non-compliance respectively begins on February 1, March 1, April 1, and

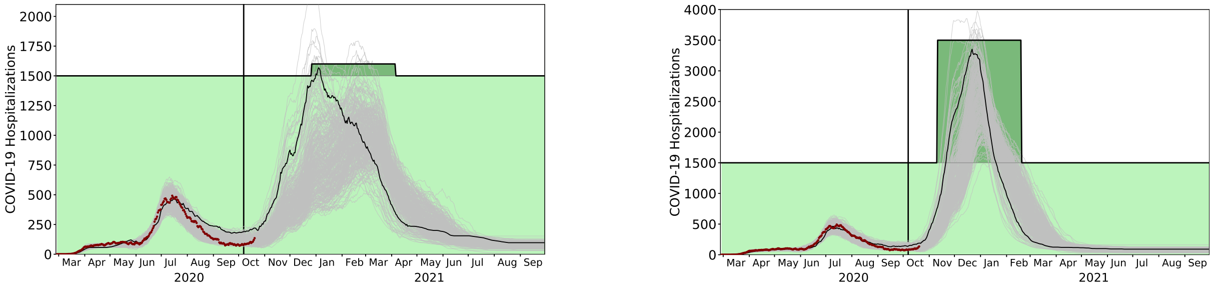

(a) (b)

Supplementary Figure 7: Projected COVID-19 hospital census for Austin under optimized alternate care site (ACS) policies, from October 7, 2020 through September 30, 2021. In both plots, the light curves indicate 300 stochastic simulations, the single solid curve is a relatively *early* projection, light green indicates the baseline COVID-19 hospital capacity, dark green indicates the timing and size of the ACS triggered by the solid curve, the red points correspond to the reported COVID-19 hospital census for all Austin area hospitals through October 20, 2020, and the vertical black line indicates the start of the projection period. (a) Under a moderate-high adherence scenario, a 100-bed ACS is launched when the seven-day rolling average of hospital admissions exceeds 220. (b) Under a low adherence scenario, a 2000-bed ACS is launched when the seven-day rolling average of hospital admissions exceeds 110. Hospital admissions are not shown.

May 1, 2021.

For Austin starting from February 1st, March 1st, and April 1st, and Houston starting from February 1st, the surge results in the 95th percentile of the number of ICU patients exceeding capacity. Detailed simulation results, which include the number of unserved patient-days and deaths follow. We compute 90% prediction intervals using symmetric order statistics, i.e., the middle 270 of 300 scenarios. As a result, the interval for unserved ICU patient-days is necessarily [0-0] whenever the 95th percentile of peak ICU demand is within capacity.

Austin:
Yellow stage starts on:

- February 1, 2021:
  - probability the number of hospitalized ICU patients exceeds COVID-19 ICU capacity: 88.7%
  - 95th percentile of peak ICU hospitalizations: 555
  - median unserved ICU patient-days: 2236
  - 90% prediction interval of unserved ICU patient-days: [0-7368]
- March 1, 2021:
  - probability the number of hospitalized ICU patients exceeds COVID-19 ICU capacity: 53.7%
  - 95th percentile of peak ICU hospitalizations: 431
  - median unserved ICU patient-days: 8
  - 90% prediction interval of unserved ICU patient-days: [0-2368]
- April 1, 2021:
  - probability the number of hospitalized ICU patients exceeds COVID-19 capacity: 5.3%
  - 95th percentile of peak ICU hospitalizations: 333
  - median unserved ICU patient-days: 0

- 90% prediction interval of unserved ICU patient-days: [0-2]

- May 1, 2021:
  - probability the number of hospitalized patients exceeds COVID-19 capacity: 2.7%
  - 95th percentile of peak ICU hospitalizations: 317
  - median unserved ICU patient-days: 0
  - 90% prediction interval of unserved ICU patient-days: [0-0]

Houston:
Yellow stage starts on:

- February 1, 2021:
  - probability the number of hospitalized ICU patients exceeds COVID-19 capacity: 74.0%
  - 95th percentile of peak ICU hospitalizations: 1294
  - median unserved ICU patient-days: 1045
  - 90% prediction interval of unserved ICU patient-days: [0-8941]
- March 1, 2021:
  - probability the number of hospitalized ICU patients exceeds COVID-19 capacity: 2.7%
  - 95th percentile of peak ICU hospitalizations: 978
  - median unserved ICU patient-days: 0
  - 90% prediction interval of unserved ICU patient-days: [0-0]
- April 1, 2021:
  - probability the number of hospitalized ICU patients exceeds COVID-19 capacity: 2.3%
  - 95th percentile of peak ICU hospitalizations: 977
  - median unserved ICU patient-days: 0
  - 90% prediction interval of unserved ICU patient-days: [0-0]
- May 1, 2021:
  - probability the number of hospitalized ICU patients exceeds COVID-19 capacity: 2.3%
  - 95th percentile of peak ICU hospitalizations: 977
  - median unserved ICU patient-days: 0
  - 90% prediction interval of unserved ICU patient-days: [0-0]

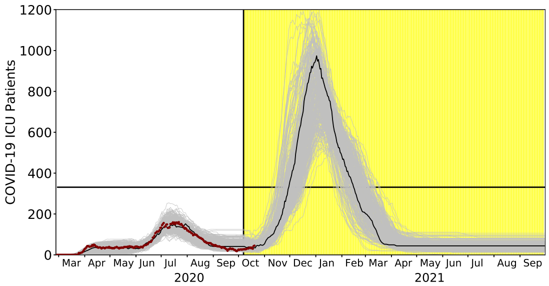

(a) ICU Hospitalizations: Austin

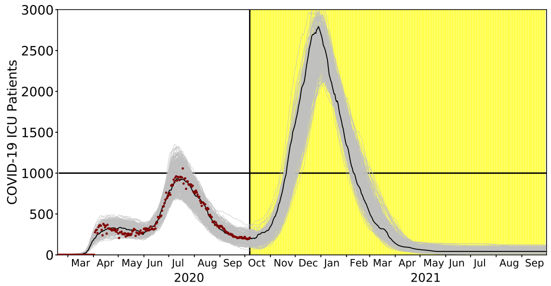

(b) ICU Hospitalizations: Houston

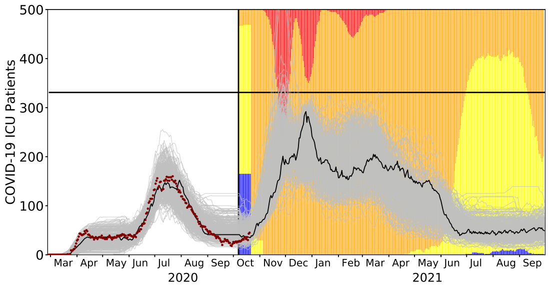

(c) ICU Hospitalizations: Austin

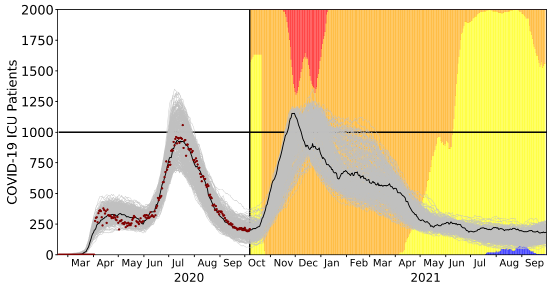

(d) ICU Hospitalizations: Houston

Supplementary Figure 8: Projections for COVID-19 intensive care unit (ICU) hospitalizations in the Austin MSA and Houston MSA when public compliance wanes, and high-risk population cocooning is not followed through the time horizon. Panels (a) and (b) present daily ICU hospitalizations (heads in beds) when the yellow stage holds after October 7th, and panels (c) and (d) present daily ICU hospitalizations when optimized triggers are used but when there is no cocooning; instead transmission reduction in the high-risk population degenerates to that of the low-risk population, with the red, orange, yellow, and blue colors representing the fraction of sample paths in each alert stage.

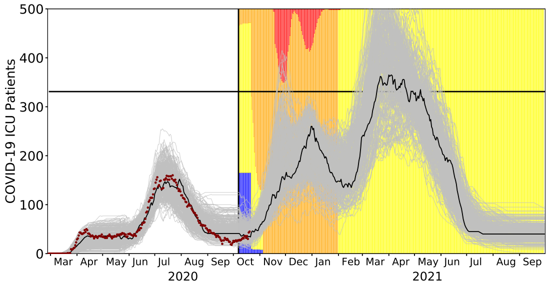

(a) Compliance until February 1st: Austin

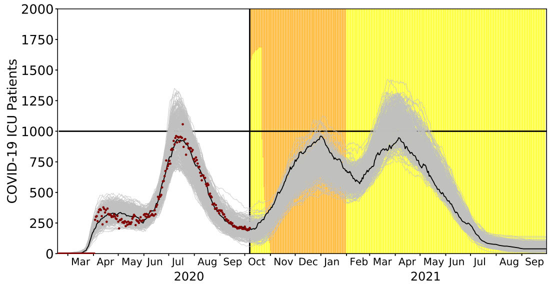

(b) Compliance until February 1st: Houston

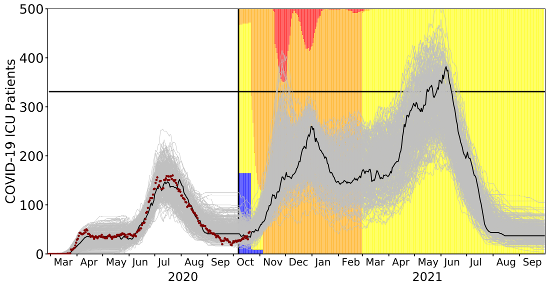

(c) Compliance until March 1st: Austin

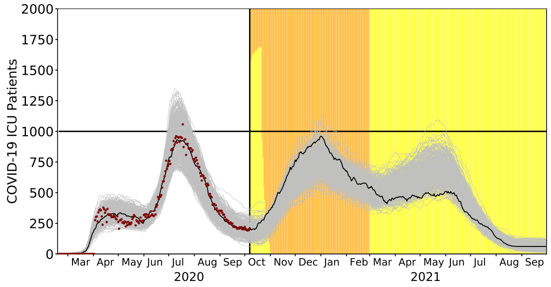

(d) Compliance until March 1st: Houston

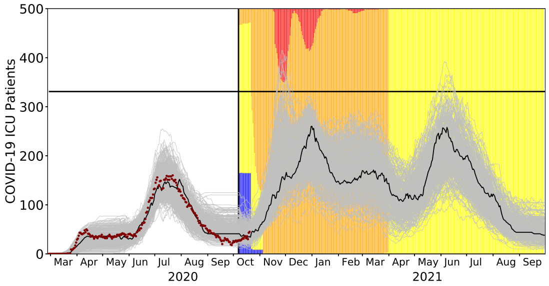

(e) Compliance until April 1st: Austin

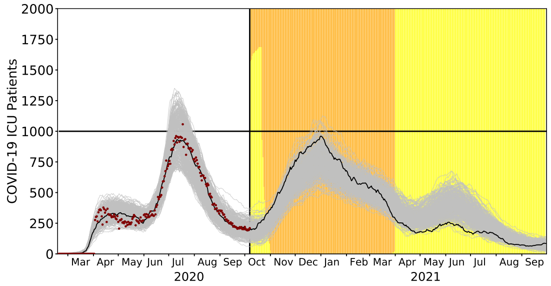

(f) Compliance until April 1st: Houston

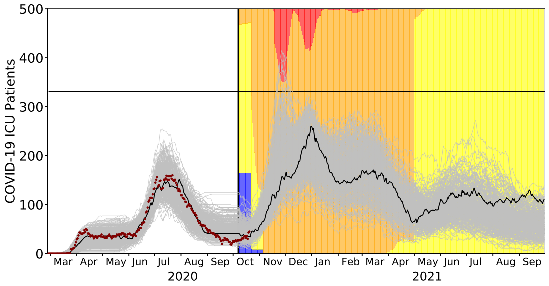

(g) Compliance until May 1st: Austin

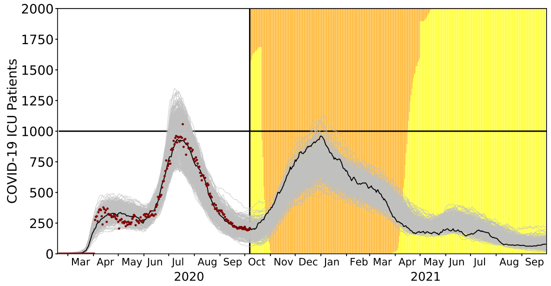

(h) Compliance until May 1st: Houston

Supplementary Figure 9: Projections for daily COVID-19 intensive care unit (ICU) hospitalizations (heads in beds) in the Austin MSA and Houston MSA when the optimal policy in Table 1 of the main text is in effect until: February 1st (a) and (b); March 1st (c) and (d); April 1st (e) and (f); and May 1st (g) and (h). The level of transmission corresponds to the yellow stage thereafter. The projections suggest that, to stay within total hospital capacity, public compliance is needed through May 1st in Austin and March 1st in Houston. The red, orange, yellow, and blue colors representing the fraction of sample paths in each alert stage

Supplementary Discussion 2. Austin's Alert System after September 30, 2020

The main manuscript includes analyses that informed policy in the Austin MSA through September 30, 2020, which includes the summer 2020 surge when the rapid growth in hospitalizations—including critical care patients—caused alarm, and when the city began planning for an alternate care site (ACS) at the convention center to handle lower acuity patients, if needed. While the beds and equipment were put in place by the end of July 2020, the ACS was in stand-by mode and not staffed—until we hit the specified trigger on January 9, 2021 [16, 17].

Following September, COVID-19 hospitalizations grew relatively slowly in Austin through mid-December and then accelerated to a peak in the middle of January 2021. Throughout this period and until today (April 2021), the City of Austin has continued to follow the staged alert system that we established in May 2020, enacting and then lifting measures when the seven-day rolling average of COVID-19 hospital admissions crossed the thresholds provided by our analyses. The city transitioned from the yellow to orange alert level on November 19, 2020, and from orange to red on December 23, 2020. The city returned to the orange alert level on February 9, 2021, and then to yellow on March 13, 2021. Throughout this period, we have continually refit the parameters of our model as new data have come available. We have revised the thresholds as area hospital estimates of ICU capacity have changed. This section briefly summarizes these more recent results, focusing on the staged alert system.

Supplementary Figure 10 shows the COVID-19 hospitalization and ICU census in the Austin, Texas MSA through April 6, 2021, with colors indicating changing alert stages throughout the period. The summer surge peaked on July 15, 2020 with 492 COVID-19 patients in area hospitals; the winter surge peaked on January 18, 2021 with 619 COVID-19 patients. ICU utilization peaked on July 22, 2020 with 160 COVID-19 patients and January 12, 2021 with 190 COVID-19 patients.

In October 2020, the major hospital systems reduced their estimate of COVID-19 ICU capacity from 331 beds to 200 beds, stemming from an increase in non-COVID patients and staffing challenges. We quickly updated our model to determine optimal triggers under this new constraint and assess if and when the policy should be updated to protect against overwhelming ICU surges. By then, the city had gone through a summer surge and several different alert levels. We fit our model to COVID-19 hospitalization throughout the spring and summer period to revise the transmission-reduction coefficients associated with each stage and then solved for optimal staging thresholds:

- red: $\kappa_1 = 0.7819$, $c_1 = 0.7819$, threshold > 50
- orange: $\kappa_2 = 0.7163$, $c_2 = 0.7437$, threshold 30-50
- yellow: $\kappa_3 = 0.6510$, $c_3 = 0.7437$, threshold 10-30
- blue: $\kappa_4 = 0.5122$, $c_4 = 0.6797$, threshold < 10.

The reduced ICU capacity (from 331 to 200 beds) led to a significantly lower (stricter) threshold for moving from the orange to red stage (a seven-day average COVID-19 hospital admission of 50 versus 120). This led city leadership to revise the thresholds in December and trigger the move to the strictest stage (red) as prescribed on December 23, 2021. As illustrated in Supplementary Figure 10, the policy led to a seven-week stay in red and narrowly prevented the ICU from surpassing local capacity.

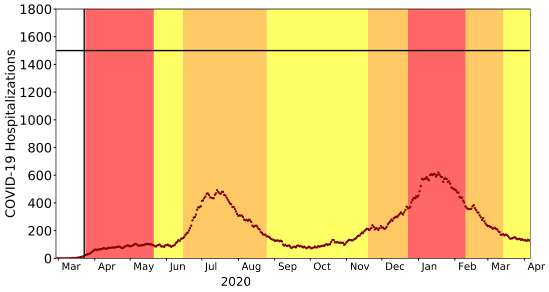

(a)

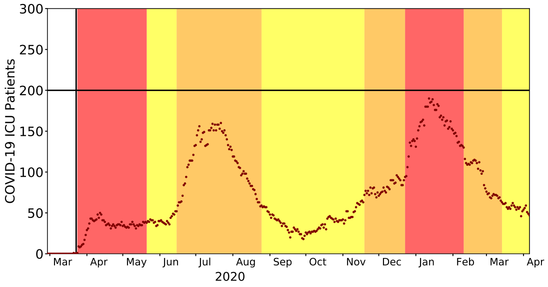

(b)

Supplementary Figure 10: COVID-19 (a) hospital census and (b) intensive care unit (ICU) census across all Austin MSA hospitals from February 28, 2020 through April 6, 2021. Background colors (red, orange and yellow) indicate the changing COVID-19 alert stage governing social distancing restrictions in the city. The horizontal black lines indicate the estimated healthcare capacity of 1500 total hospital beds and 200 ICU beds.

Parameters	Values	Source
β: transmission rate	Austin: 0.06901<br>Houston: 0.06401	[5, 18]
P: proportion of pre-symptomatic transmission (%)	44	[6]
ω_A: infectiousness of individuals in compartment IA, relative to IY	$\omega_A \sim$ Triangular (0.29,0.29,1.4)	[19]
τ : symptomatic proportion (%)	57	[20]
ω_P: infectiousness of individuals in pre-symptomatic and pre-asymptomatic compartments, relative to symptomatic and asymptomatic compartments	$\omega_P = \frac{P}{1-P} \frac{\tau(\frac{YHR}{\eta_H}+\frac{1-YHR}{\gamma_Y})+(1-\tau)\frac{\omega_A}{\gamma_A}}{\frac{\tau}{\rho_Y}+(1-\tau)\frac{\omega_A}{\rho_A}}$	
σ : exposed rate	$\frac{1}{\sigma} \sim$ Triangular (1.9, 2.9, 3.9)	Based on incubation [21] and pre-symptomatic periods
γ_A: recovery rate from compartment IA	$\frac{1}{\gamma_A} \sim$ Triangular (3, 4, 5)	[6]
γ_Y: recovery rate from symptomatic compartment IY	$\frac{1}{\gamma_Y} \sim$ Triangular (3, 4, 5)	[6]
ρ_A: rate at which pre-asymptomatic individuals become asymptomatic	Equal to ρ_Y	[6]
ρ_Y: rate at which pre-symptomatic individuals become symptomatic	$\frac{1}{\rho_Y} = 2.3$	[6]
IFR: infected fatality ratio, age specific (%)	Low risk / High risk<br>0.000917 / 0.00917<br>0.00218 / 0.0218<br>0.0339 / 0.339<br>0.252 / 2.52<br>0.644 / 6.44	Age adjusted from [22]
YFR: symptomatic fatality ratio, age specific (%)	Low risk / High risk<br>0.00161 / 0.0161<br>0.00382 / 0.0382<br>0.0594 / 0.594<br>0.442 / 4.42<br>1.13 / 11.3	$YFR = \frac{IFR}{1-\tau}$
C: daily cost to stay at specific stages	C_1 1000<br>C_2 100<br>C_3 10<br>C_4 1	

Supplementary Table 10: Model parameters

Parameters	Value	Source
η_H : rate from symptom onset to hospital admission	0.1695	5.9 day average from symptom onset to hospital admission [23]
YHR: symptomatic case hospitalization rate (%)	Low risk / High risk<br>0.0279 / 0.2791<br>0.0215 / 0.2146<br>1.3215 / 13.2514<br>2.8563 / 28.5634<br>3.3873 / 33.8730	Age adjusted from [22]
p_{IH}	Fitted time series, starting at 0.6717	Hospital system data
γ_H, γ_{ICU} : recovery rate in compartment IH and ICU	Fitted parameters	Hospital system data
π : rate symptomatic individuals go to hospital, age-specific	$\pi = \frac{\gamma_Y \cdot YHR}{\eta_H + (\gamma_Y - \eta_H) YHR}$	
η_{ICU} : rate from hospital admission to ICU	A time series which is constant specific to time blocks	Hospital system data
μ : rate from ICU to death	Fitted parameter	Hospital system data
$ICUFR$: ICU death ratio, age specific (%)	$ICUFR$<br>5.8592<br>5.8592<br>5.8592<br>15.6207<br>30.8526	Hospital system data
$HICUR$: hospitalized ICU ratio	A time series with a decreasing rate specific to time blocks, starting at 0.1574	Hospital system data
ν_H : ICU rate on hospitalized individuals, age-specific	$\nu_H = \frac{\gamma_H \cdot HICUR}{\eta_{ICU} + (\gamma_H - \eta_{ICU}) HICUR}$	
ν_{ICU} : death rate on ICU individuals, age-specifc	$\nu_{ICU} = \frac{\gamma_{ICU} \cdot ICUFR}{\mu + (\gamma_{ICU} - \mu) ICUFR}$	
B: Total hospital bed capacity (including ICU)	Austin: 1500<br>Houston: 4500	Estimates provided by each of the region's hospital systems and aggregated by regional public health leaders
B_{ICU}: ICU capacity	Austin: 331<br>Houston: 1250	Estimates provided by each of the region's hospital systems and aggregated by regional public health leaders
$1_{\{\text{school closure}\}}$: school closure dates	Austin: $3/19/2020 - 9/8/2020$, $5/26/2021 - 8/23/2021$<br>Houston: $3/19/2020 - 9/8/2020$, $5/28/2021 - 8/23/2021$	

Supplementary Table 11: Hospitalization parameters

Supplementary References

[1] Austin Public Health (APH). COVID-19: Risk-based guidelines (2020). URL http://austintexas.gov/sites/default/files/files/Health/Risk-Based-Guidance%20Eng-v14.pdf.

[2] Harris County Public Health. Leading Harris county: Public guidelines for a healthy community (2020). URL https://www.readyharris.org/Portals/60/documents/INFO_ReopeningStrategyPUBLIC-FULL.pdf.

[3] Rathouz, P. J. *et al.* Survival analysis methods for analysis of hospitalization data: Application to COVID-19 patient hospitalization experience. *medRxiv* (2021). URL https://www.medrxiv.org/content/early/2021/04/20/2021.04.14.21255511.

[4] Wang, X. *et al.* Impact of social distancing measures on coronavirus disease healthcare demand, central texas, USA. *Emerging Infectious Diseases* **26**, 2361–2369 (2020).

[5] Duque, D. *et al.* Timing social distancing to avert unmanageable COVID-19 hospital surges. *Proceedings of the National Academy of Sciences* (2020).

[6] He, X. *et al.* Temporal dynamics in viral shedding and transmissibility of COVID-19. *Nature Medicine* **26**, 672–675 (2020).

[7] Centers for Disease Control and Prevention. 500 cities: Local data for better health (2019). URL https://www.cdc.gov/500cities/definitions/health-outcomes.htm.

[8] The Mayor of the City of Austin. STAY HOME – WORK SAFE ORDER NO. 20200413-009, The City of Austin (2020). URL http://www.austintexas.gov/sites/default/files/files/document_96DEBEEC-E581-05E0-8A3D444404948A84.pdf.

[9] Harris County. Stay home, work safe (2020). URL https://www.readyharris.org/Newsroom/News-Releases/All-News-Releases/harris-county-issues-stay-home-work-safe-order.

[10] The Governor of Texas. Executive Order GA18: Relating to the expanded reopening of services as part of the safe, strategic plan to open Texas in response to the COVID-19 disaster (2020). URL https://gov.texas.gov/uploads/files/press/EO-GA-18_expanded_reopening_of_services_COVID-19.pdf.

[11] The Governor of Texas. Executive Order GA28: Relating to the targeted response to the COVID-19 disaster as part of the reopening of Texas (2020). URL https://gov.texas.gov/uploads/files/press/EO-GA-28_targeted_response_to_reopening_COVID-19.pdf.

[12] The Mayor of the City of Austin. Order 20200626-016: Stay home, mask, and otherwise be safe (2020). URL https://www.austintexas.gov/sites/default/files/files/Health/Order%20No.%2020200626-016-StayHome-Mask-Otherwise-Be-Safe.pdf.

[13] Virtanen, P. *et al.* SciPy 1.0: Fundamental Algorithms for Scientific Computing in Python. *Nature Methods* (2020).

[14] Ministère des Solidarités et de La Sante. Point hebdomadaire COVID-19 (2020). URL https://www.gouvernement.fr/sites/default/files/cfiles/prez_-_conf_de_presse_23092020_-_vdef.pdf.

[15] Harvard Global Health Institute. Key metrics for COVID suppression (2020). URL https://globalepidemics.org/wp-content/uploads/2020/09/key_metrics_and_indicators_v5-1.pdf.

[16] City of Austin. Austin-Travis County begins work to open alternate care site (2021). URL https://www.austintexas.gov/news/austin-travis-county-begins-work-open-alternate-care-site.

[17] City of Austin. Austin-Travis County alternate care site ready to take patients (2021). URL https://www.austintexas.gov/news/austin-travis-county-alternate-care-site-ready-take-patients.

[18] Yang, H. *et al.* Staged strategy to avoid hospital surge and preventable mortality, while reducing the economic burden of social distancing measures. Tech. Rep., The University of Texas at Austin (2020).

[19] He, D. *et al.* The relative transmissibility of asymptomatic cases among close contacts. *International Journal of Infectious Diseases* **94**, 145–147 (2020).

[20] Gudbjartsson, D. F. *et al.* Spread of SARS-CoV-2 in the Icelandic population. *New England Journal of Medicine* **382**, 2302–2315 (2020).

[21] Lauer, S. A. *et al.* The incubation period of coronavirus disease 2019 (COVID-19) from publicly reported confirmed cases: Estimation and application. *Annals of Internal Medicine* (2020).

[22] Verity, R. *et al.* Estimates of the severity of coronavirus disease 2019: a model-based analysis. *The Lancet Infectious Diseases* **20**, 669–677 (2020).

[23] Tindale, L. *et al.* Transmission interval estimates suggest pre-symptomatic spread of COVID-19. *Preprint* (2020).
