# Supplementary material for: Design of COVID-19 staged alert systems to ensure healthcare capacity with minimal closures
Source: Nat Commun. 2021 Jun 18;12:3767. doi: 10.1038/s41467-021-23989-x (PMC8213780; doi:10.1038/s41467-021-23989-x)
Supplement: Supplementary file 3 — Reporting Summary [file 41467_2021_23989_MOESM3_ESM.pdf]

nature research

Corresponding author(s): David P. Morton
Last updated by author(s): May 13, 2021

Reporting Summary

Nature Research wishes to improve the reproducibility of the work that we publish. This form provides structure for consistency and transparency in reporting. For further information on Nature Research policies, see our Editorial Policies and the Editorial Policy Checklist.

Statistics

For all statistical analyses, confirm that the following items are present in the figure legend, table legend, main text, or Methods section.

n/a	Confirmed	
☐	☒	The exact sample size (n) for each experimental group/condition, given as a discrete number and unit of measurement
☐	☒	A statement on whether measurements were taken from distinct samples or whether the same sample was measured repeatedly
☒	☐	The statistical test(s) used AND whether they are one- or two-sided<br>*Only common tests should be described solely by name; describe more complex techniques in the Methods section.*
☒	☐	A description of all covariates tested
☐	☒	A description of any assumptions or corrections, such as tests of normality and adjustment for multiple comparisons
☒	☐	A full description of the statistical parameters including central tendency (e.g. means) or other basic estimates (e.g. regression coefficient) AND variation (e.g. standard deviation) or associated estimates of uncertainty (e.g. confidence intervals)
☒	☐	For null hypothesis testing, the test statistic (e.g. F, t, r) with confidence intervals, effect sizes, degrees of freedom and P value noted<br>*Give P values as exact values whenever suitable.*
☐	☒	For Bayesian analysis, information on the choice of priors and Markov chain Monte Carlo settings
☒	☐	For hierarchical and complex designs, identification of the appropriate level for tests and full reporting of outcomes
☒	☐	Estimates of effect sizes (e.g. Cohen's d, Pearson's r), indicating how they were calculated

Our web collection on statistics for biologists contains articles on many of the points above.

Software and code

Policy information about availability of computer code

Data collection	Data were collected by area hospitals and reported to us via encrypted email as CSV files.
Data analysis	Our analysis is built atop a high-fidelity SEIR (susceptible-exposed-infectious-recovered) epidemiological simulation model, which is implemented in custom code. The parameters of that SEIR model are specified in the paper's Appendix or are computed via a least-squares fit of the model to hospitalization data. The open-source optimization software used to perform the fit is the SciPy 1.4.1 / Python 3 routine scipy.optimize.least_squares using the trust region reflective algorithm (trf), and it is employed as described in the Appendix C. The pseudo-code given in Algorithm 1 of Appendix C is custom code implemented in Python. Finally, the grid search used to optimize model [3] in Appendix A is implemented in custom Python 3 code. The codes described here are all available at the repository: https://github.com/haoxiangyang89/COVID_Staged_Alert.

For manuscripts utilizing custom algorithms or software that are central to the research but not yet described in published literature, software must be made available to editors and reviewers. We strongly encourage code deposition in a community repository (e.g. GitHub). See the Nature Research guidelines for submitting code & software for further information.

Data

Policy information about availability of data

All manuscripts must include a data availability statement. This statement should provide the following information, where applicable:
- Accession codes, unique identifiers, or web links for publicly available datasets
- A list of figures that have associated raw data
- A description of any restrictions on data availability

Area hospitals in the Austin, Texas MSA collected and reported the following data: (i) census data on total COVID-19 patients hospitalized each day, (ii) census data on number of COVID-19 patients in the ICU each day, and (iii) admissions of COVID-19 patients to the general ward or ICU each day. These data can be found at the

repository: https://github.com/haoxiangyang89/COVID_Staged_Alert. These data are used to fit key SEIR model parameters as described in Appendix C. Our analysis also uses data on the length of COVID-19 patient durations in the general ward and the ICU, as well as data on the proportion of hospitalized patients that are admitted to the ICU. That data came from a multi-facility hospital system serving the Central Texas region, including Austin, Texas. The use of these data to generate the input model parameters (reported in Appendix C) was approved as exempt human subjects research by the IRB of the University of Texas at Austin. The remaining data used in our analysis are also available at the GitHub repository and further made available and described in the Appendix.

Field-specific reporting

Please select the one below that is the best fit for your research. If you are not sure, read the appropriate sections before making your selection.

☐ Life sciences ☒ Behavioural & social sciences ☐ Ecological, evolutionary & environmental sciences

For a reference copy of the document with all sections, see nature.com/documents/nr-reporting-summary-flat.pdf

Behavioural & social sciences study design

All studies must disclose on these points even when the disclosure is negative.

Study description	The study uses an SEIR epidemiological simulation model of COVID-19 spread in the Austin, Texas metropolitan statistical area (MSA) to guide community mitigation strategies, using optimized, quantitative triggers to toggle between stages of an alert system.
Research sample	Key data used to inform the study include admissions and census data for Austin MSA hospitals. The data were reported for all hospitals in the Austin, Texas MSA, and in this sense the data are exhaustive because the study is restricted to this MSA. Policy decisions regarding institution and relaxation of community mitigation strategies are performed at the level of the city's jurisdiction.
Sampling strategy	COVID-19 hospitalization data were reported on a daily basis for the duration of the pandemic to-date and in this sense are exhaustive. The sampling procedure consisted of Monte Carlo sampling for specified distributions, both for macro and micro stochastics. The former concern key uncertain parameters in the SEIR model and the latter involving binomially distributed number of people transitioning between compartments in the SEIR model. Algorithm 1 in the Appendix provides pseudo-code for the sampling procedure. The analysis uses 300 Monte Carlo sample paths. To assess whether this sample size was sufficiently large, we compared the consistency of our estimates (e.g., probability of violating ICU capacity) between in-sample results (in which trigger thresholds were selected based on a specific set of 300 sample paths) and out-of-sample Monte Carlo samples paths (assessment using the optimized trigger thresholds under a new independent set of 300 sample paths).
Data collection	COVID-19 hospitalization data were collected, stored, and reported electronically by computer from individual hospitals, and hospital systems, and are used in aggregate in our study. The data were not collected by the researchers of the study.
Timing	The SEIR simulation model is seeded on February 28, 2020 for Austin and February 19, 2020 for Houston. These dates are two weeks prior to the first confirmed COVID-19 cases in each city.
Data exclusions	No data were excluded.
Non-participation	Our study did not involve recruiting or reporting from individual participants, but rather involved aggregate reporting of hospitalization data.
Randomization	Our study did not involve randomizing participants or require controlling covariates. In our simulation analysis, we drew random sample paths of how the pandemic progresses in the Austin, Texas MSA.

Reporting for specific materials, systems and methods

We require information from authors about some types of materials, experimental systems and methods used in many studies. Here, indicate whether each material, system or method listed is relevant to your study. If you are not sure if a list item applies to your research, read the appropriate section before selecting a response.

Materials & experimental systems

n/a	Involved in the study	
☒	☐	Antibodies
☒	☐	Eukaryotic cell lines
☒	☐	Palaeontology and archaeology
☒	☐	Animals and other organisms
☒	☐	Human research participants
☒	☐	Clinical data
☒	☐	Dual use research of concern

Methods

n/a	Involved in the study	
☒	☐	ChIP-seq
☒	☐	Flow cytometry
☒	☐	MRI-based neuroimaging
